# Supplementary material for: A post-Brexit intergroup contact intervention reduces affective polarization between Leavers and Remainers short-term
Source: Commun Psychol. 2024 Oct 14;2:95. doi: 10.1038/s44271-024-00146-w (PMC11473807; doi:10.1038/s44271-024-00146-w)
Supplement: Supplementary file 2 — Supplementary Material [file 44271_2024_146_MOESM2_ESM.pdf]

Supplementary Information

A post-Brexit intergroup contact intervention reduces affective polarization between Leavers and Remainers short-term

Nicole Tausch*[1], Michèle D. Birtel[2], Paulina Górska[3], Sidney Bode[1] and Carolina Rocha[4]

[1]University of St Andrews, School of Psychology and Neuroscience, St Andrews, UK
[2]University of Greenwich, Institute for Lifecourse Development, School of Human Sciences, London, UK
[3]University of Warsaw, Faculty of Psychology, Warsaw, Poland
[4]University of Dundee, School of Business, Dundee, UK

*Corresponding Author: Nicole Tausch, School of Psychology and Neuroscience, St Mary's Quad, South Street, St Andrews, KY16 9JP; Email: nt20@st-andrews.ac.uk.

Supplementary Methods

Supplementary Methods 1

Participant recruitment. Our initial call for participants was posted on a number of Facebook sites related to Brexit and UK politics more generally, representing a range of political views (e.g., Brexit means Brexit, Remainers United, UK Politics Debate, Politics & Beyond). Through this method we obtained an initial sample of 163 (16 Leavers, 147 Remainers) who completed the T1 (pre-intervention) survey. Of these respondents, 114 (69.9%) indicated that they would like to continue with the study. Participants were contacted via email to arrange a timeslot for the interaction phase. Four participants (2 Leavers, 2 Remainers) successfully completed the interaction and remaining phases of the experiment. The remaining participants either did not respond further (N = 8), or, due to the unbalanced nature of this sample, could not be assigned an interaction partner.

A second phase of recruitment was conducted via Prolific. We advertised the study to Prolific users who had voted in the 2016 referendum, either supporting or opposing Brexit, and who indicated that they had a webcam and that they were willing to participate in a face-to-face video call. We received T1 data from 423 participants (176 Leavers, 247 Remainers) via this route. Of this sample, 370 participants indicated that they would like to participate in the remaining phases of the study. We used the Prolific messaging service to communicate with participants to arrange the interaction sessions. One-hundred and sixteen participants successfully completed all phases of the experiment. The remaining participants either did not reply to our messages, did not confirm their interaction slot, or did not show (N=164), or were unable to find a suitable interaction slot (N=90).

We scheduled two participants, one Leaver and one Remainer, for each experimental session. In case a participant did not show, the other participant was sent home and the session was rescheduled, or, where necessary (i.e., when the original interaction partner did not respond to our messages), they were assigned a new interaction partner.

Supplementary Methods 2

Intervention sample details. Supplementary Table 1a provides an overview of the demographic characteristics of our intervention sample (*N* = 120).

Supplementary Table 1a. Demographic characteristics of participants by group and condition.

Condition	Contact Intervention		Control	
Group	Leaver	Remainer	Leaver	Remainer
N	31	31	29	29
Gender				
Female	14	20	17	16
Male	17	11	12	13
Age				
Range (24-79)	47.48 $\pm$ 14.29	41.55 $\pm$ 12.49	46.17 $\pm$ 12.47	44.43 $\pm$ 13.28
Education level				
Second. school	2	0	1	0
GCSE	4	2	5	0
A-level	10	8	7	4
Undergraduate	9	13	13	14
Postgraduate	6	8	3	11
Ethnicity				
White	29	25	27	25
Black	1	3	1	1
Asian	0	1	0	2
Mixed	0	1	0	0
Missing	-	-	1	1
Residence				
England	28	24	26	25
Scotland	3	4	0	2
Wales	0	2	2	2
Norther Ireland	0	0	0	0
Other	0	1	1	0

Note. Age reported as mean $\pm$ standard deviation.

Political party support in the intervention sample by Brexit opinion group. In accordance with prior survey results, which indicated that partisanship and political orientation and Brexit identities are correlated (e.g., Conservatives (60%) and people on the political right (48%) were more likely to vote Leave. Liberal Democrats (70%), Labour (53%) and people on the political left (66%) were more likely to vote Remain; Pew Research Center, 2019), our data show an alignment between partisanship and Brexit position. Leavers reported more frequently voting for the Conservatives, Remainers reported more frequently voting for Labour or the Liberal Democrats (see Supplementary Table 1b).

Supplementary Table 1b. Past voting and voting intentions in the UK general election, by group

		Remain	Leave
2015 General Election	Conservatives	28.3	68.3
	Labour	46.7	13.3
	Liberal Democrats	15	0
2019 General Election	Conservatives	20	75
	Labour	46.7	10
	Liberal Democrats	13.3	1.7
Next General Election	Conservatives	20	61.7
	Labour	43.3	13.3
	Liberal Democrats	10	1.7

Items: *Which political party did you vote for in the 2015 / 2019 general election? Which political party would you vote for in the next general election?*

Supplementary Methods 3

Ground rules. A set of ground rules for the interaction relating to privacy and confidentiality of the online interaction, respectful conduct during the interaction, and potential discomfort were emailed to participants in the days prior to the scheduled interaction.

Supplementary Table 2. *Ground rules provided via email prior to the interaction.*

**Privacy and confidentiality**<br>You will participate in a video interaction on Microsoft Teams with another participant. A moderator will be present during this time to guide you through the various topics of discussion and to ensure everyone is feeling comfortable. To protect your privacy, we suggest that you do not disclose your full name, place of residence or work place during the interaction. You can join the video interaction by choosing your first name or a nick name by which you would like to be addressed during the interaction. The interaction is not to be recorded by anyone, neither the moderator not the participants, and we ask you to not to share any information revealed by your interaction partner with others. Please use a private and quiet location to ensure that the meeting is not interrupted and that the discussion is not overheard by others.<br><br>**Respectful dialogue**<br>You will be asked to engage in a dialogue with another participant, in which we aim to explore each other's perspectives, thoughts and feelings. This will not a debate, we do not seek for a winner, and it is not a discussion in which we want to persuade or impose one view over another. The aim is to acknowledge different perspectives, and listen in order to understand, not in order to judge or discount the other person's thoughts and feelings. We want you to listen carefully and respectfully to each other. Please phrase your questions and comments in a way that does not imply that you think the other person's opinion is wrong by, for example, using "I" messages, such as, "I feel strongly...".<br><br>**What if you feel uncomfortable**<br>If you feel uncomfortable, need a break and/or wish to terminate the session early, please do not hesitate to say so or contact the moderator by sending them a private chat message if you wish.

Interaction protocol. The interaction consisted of an introduction, a personal acquaintance task (Stage 1), a respectful exchange of views on Brexit (experimental group) or the Royal family and "Megxit" (control group) (Stage 2), a cooperative task (Stage 3), and a conclusion.

Supplementary Table 3. *Interaction protocol used by moderators.*

Introduction (˜5 minutes)<br>*Moderator introduces themselves by name.*<br>My role as moderator is to facilitate the discussion, act as a guide, and provide support. I take a neutral position on the issues discussed and I will not comment or provide an opinion on the topic or any comments made by you. This session will take around 40-60 minutes and consist of 3 stages that will have different conversation topics. I will guide you through each stage. Before we begin I want to remind you of the ground rules for this conversation that have been emailed to you: [summarizes the Ground Rules emailed to participants] Do you have any questions so far? Are you happy to continue?

Stage 1: Personal acquaintance task (de-categorization) (˜10 minutes)

Let's start with introducing yourselves to each other. Things you could share: your first name or nickname, which area of the UK you are from, your hobbies.
To warm up, we want you to answer four questions about yourself that we have prepared for you. You will take turns in answering questions.

1. Given the choice of anyone in the world, whom would you want as a dinner guest?
2. What would constitute a "perfect" day for you?
3. For what in your life do you feel most grateful?
4. What do you value most in a friendship?

Stage 2: Respectful intergroup dialogue (categorization) (˜15-30 minutes)

2.1 Setting ground rules and creating a shared meaning of dialogue

This part is a dialogue in which we aim to explore each other's perspectives, thoughts and feelings. Each of you will have time to share their view. Please do not interrupt each other but listen carefully, seeking to understand rather than to counterargue. A good method to actively listen is to repeat the other person's words in your own words, and to ask questions – before stating your own view. Every participant is responsible of creating a relaxed and safe environment to exchange perspectives.

2.2a Experimental group: Exchange views on Brexit.

In this part we would like you to discuss the issue of Brexit. Specifically, we would like you to share your view on Brexit and explain why you have this particular position on Brexit. You could start for example, by telling us how you voted in the 2016 EU referendum and why you voted this way. Do you have any questions so far? Are you happy to continue?

If the conversation dries up, use some of these prompts:

1. What are your views on the European Union?
2. What are the advantages and disadvantages of Brexit?
3. How do you think will Brexit impact on people's lives?
4. What are the most important issues in the UK right now for you?
5. How should the country move forward now that Brexit has happened?

2.2b Control group: Exchange views on the Royal Family.

In this part we would like you to discuss your views on Prince Harry's and Meghan Markle's exit from the Royal Family, and explain why you have this particular position. You could start for example, by telling us how you felt when you heard of Meghan and Harry's decision. Do you have any questions so far? Are you happy to continue?

If the conversation dries up, use some of these prompts:

1. What are your views on the Royal Family?
2. What are the advantages and disadvantages of Meghan and Harry's exit?
3. How do you think this decision will impact on people's lives?
4. What are the most important issues for the Royal Family right now for you?
5. How can the Royal family move forward after Meghan and Harry's exit?

Stage 4: Cooperative contact toward a superordinate goal (re-categorization) (˜10 minutes)<br>Finally, we would like to discuss some broader issues. We are interested in people's views about the mental and physical wellbeing of people in the UK during the pandemic. What could local councils do to improve people physical and mental wellbeing during the pandemic? We would like you to ask you to work together and brainstorm to come up with some concrete actions that should be taken. I (the moderator) will take notes of your proposed actions. These will be anonymous and cannot be linked to your identity. We will collate suggestions from all participants in this study and submit a summary of suggestions to the Parliamentary Office of Science and Technology team, for consideration by policy advisers.<br><br>Do you have any questions? Are you happy to continue?
Conclusion (˜10 minutes)<br>*Thank participants and direct them to the online questionnaire. Check if they have any questions about the remainder of the study.*

Supplementary Methods 4

Unless otherwise indicated, the measures were administered at all three time points. All items gave participants the opportunity to omit an answer by selecting 'prefer not to answer'. 'Prefer not to answer' responses were treated as missing values. Correlations between all variables are reported in Table 37 available on the OSF project website (https://osf.io/ra6yj/).

(1) Background variables and Brexit position

Demographic variables. At T1, participants indicated their age (in numbers), their gender (female, male, non-binary, prefer to self-describe), highest education level completed (1 = no formal schooling, 2 = primary school, 3 = secondary school, 4 = GCSE or similar, 5 = A-level or similar, 6 = undergraduate education, 7 = postgraduate education), and their country of residence (England, Scotland, Wales, Northern Ireland, other). At T3, participants further indicated their ethnicity, using the following categories: White (English/Welsh/Scottish/Northern Irish/British; Irish; Gypsy or Irish Traveller; other White background), Mixed (White and Black Caribbean; White and Black African; White and Asian; other Mixed/multiple ethnic background), Asian/Asian British (Indian; Pakistani; Bangladeshi; Chinese; other Asian background), Black/Black British (African; Caribbean; other Black/African/Caribbean British background), Arab, Latinx/Hispanic, other ethnic group.

Political orientation and voting. Political orientation was measured at T3 through the item "As you may know, generally people define political orientations in terms of being on the left, the centre or the right. Please use the scale below to indicate your political orientation" on a 7-point Likert scale ranging from 1 (*left*) to 7 (*right*). At T3, participants also indicated their votes in the 2015 and 2019 UK general election, as well as which political party they would vote for in the next general election (Conservatives; Labour; Liberal Democrats; SNP; UKIP/Brexit Party/UK Reform; other [please specify]; I did/would

not vote). Identification with the part selected for their next election vote was measured through five items (based on Cameron, 2004): “I have a lot in common with other [party] supporters”; “Being a [party] supporter is an important part of how I see myself”; “I feel personally criticised when someone criticises [party] supporters”; “I feel strong ties with other [party] supporters”; “I'm proud to be a [party] supporter”. The items were combined into an index of party identification (α = .89).

Brexit stance. At T1, participants indicated their vote in the 2016 Brexit referendum (Leave, Remain, did not vote). Participants also indicated whether they currently identify as either Leaver or Remainer (all three time points). Only participants who indicated to have voted in the 2016 referendum and who currently identified as either Leaver or Remainer were allowed to continue with the study.

Identification as Leaver/Remainer. Level of identification as Leaver or Remainer was measured at T1 through five items “I have a lot in common with other Leavers/Remainers”; “Being a Leaver/Remainer is an important part of how I see myself”; “I feel personally criticised when someone criticises Leavers/Remainers”; “I feel strong ties with other Leavers/Remainers”; “I'm proud to be a Leaver/Remainer”. The items were combined into an index of identification (α = .84).

(2) Outgroup contact quantity and quality

We assessed quantitative and qualitative aspects of everyday contact with members of the outgroup (i.e., Leavers for self-identified Remainers, Remainers for self-identified Leavers) at T1 and T3.

Outgroup contact (social networks). At T1, participants indicated how many of their *acquaintances*, *close friends*, and *family members* are outgroup members (1 = none, 2 = a few, 3 = about half, 4 = more than half, 5 = most, 6 = all, don’t know). We combined responses into an overall index of social network penetration (α = .80).

Outgroup contact (overall frequency). A single item was used to assess overall contact frequency with outgroup members. At T1, participants indicated how frequently they (knowingly) interacted with outgroup members over the last few months (1 = *never*, 2 = *rarely*, 3 = *occasionally*, 4 = *often*, 5 = *very often*). At T3, participants answered the same question with respect to the time period in the past month (i.e., since the intervention).

Outgroup contact quality. At T1 and T3 participants indicated to what extent their contact with outgroup members was overall *respectful* (1 = extremely disrespectful, 7 = extremely respectful), *friendly* (1 = extremely unfriendly, 7 = extremely friendly), *comfortable* (1 = extremely uncomfortable, 7=extremely comfortable), and *equal* (1 = extremely unequal, 7 = extremely equal). The items were combined to form a composite index of contact quality (α_{T1} = .85; α_{T3} = .90).

(3) Perceptions of the interaction

Directly following the interaction, participants indicated their current affect and rated the interaction in terms of overall quality, equality-based respect, and perceived similarity to the interaction partner.

Post-interaction state affect. We measured how participants felt directly after participating in the interaction at T2 using the ten items of the Positive and Negative Affect Schedule (PANAS, Watson et al., 1988). On a 5-point Likert scale ranging from 1 = *not at all*

to 5 = *very much,* participants indicated the extent to which they felt *inspired*, *alert*, *excited*, *enthusiastic*, *determined*, *afraid*, *upset*, *nervous*, *scared*, and *relieved* at this moment. Items were presented in a random order. With exception of *relieved*, which was excluded from further analysis, the items loaded a factor indicating positive affect (*inspired*, *alert*, *excited*, *enthusiastic*, *determined*; Eigenvalue = 3.19, 31.85% explained variance) and negative affect (*afraid*, *upset*, *nervous*, *scared*, Eigenvalue = 2.32, 23.18% explained variance). The items were combined to form indices of positive ($\alpha_{positive}$ = .84) and negative ($\alpha_{negative}$ = .67) state affect.

Perceived quality of interaction. To assess the experienced quality of the interaction, participants indicated the extent to which they felt that the interaction was *positive* (1=extremely negative, 7=extremely positive); *respectful* (1=extremely disrespectful, 7=extremely respectful), *friendly* (1 = extremely unfriendly, 7 = extremely friendly), *comfortable* (1=extremely uncomfortable, 7 = extremely comfortable), and *equal* (1 = extremely unequal, 7 = extremely equal). The items were combined into an index of quality of the interaction (α = .85).

Equality-based respect *during* the interaction. We assessed equality-based respect experienced during the interaction with six items adapted from Renger and Reese (2017): "My interaction partner communicated with me as with a person of equal worth", "All in all, my interaction partner definitely treated me as a bearer of equal rights", "My interaction partner always treated me as a human being with equal ability", "I was consistently treated as a counterpart that is to be taken seriously", "My interaction partner treated me as an intelligent person." "My interaction partner treated me as a decent person". The items were combined into a composite indicating felt equality-based respect during the interaction (α = .90).

Perceived similarity of interaction partner. Participant were asked to rate the extent to which they thought that there are similarities or differences between them and their interaction partner in terms of: values, everyday concerns, and strength of community ties (α = .73).

(4) Focal outcome measures

To assess different aspects of affective polarization, we administered a battery of measures consisting of adaptations of established measures of outgroup warmth, outgroup emotions, stereotypes, meta-beliefs, attributions, compromise willingness and collective action intentions. We also created novel scales to capture key aspects of outgroup contempt and Brexit beliefs. All of the below scales were administered at all three time points.

Outgroup warmth. Warmth towards the outgroup was measured on a feeling thermometer ranging from 0 to 100. Responses were given on a slider with the following instruction: How do you feel toward [outgroup members]? Please rate [outgroup members] on a thermometer that runs from zero (0) to a hundred (100) degrees. The higher the number, the warmer or more favourable you feel towards [outgroup members]. The lower the number, the colder or less favourable you feel. If you feel neither warm nor cold towards [outgroup members], rate them at 50.

Outgroup emotions. On scales ranging from 1 (*not at all*) to 7 (*extremely*), participants indicated to what extent they felt the following emotions toward [outgroup members]: anger, disgust, contempt, respect (R). The 'respect' item was reversed and all

items were then combined into reliable indices of negative emotions toward the outgroup (α_{T1} = .79; α_{T2} = .81; α_{T3} = .79).

Outgroup stereotypes. To assess outgroup stereotypes, we asked participants: "To what extent do you think [outgroup members] possess the following traits: intelligent, intolerant". Answer scales ranged from 1 (*not at all*) to 7 (*very much*). Correlations between the two items were moderate (r= -.24 at T1, r = -.27 at T2, r= -.35 at T3) and we analysed the items separately.

Meta-emotions. To assess meta-emotions (see Pauketat, Mackie, & Tausch, 2020) participants were asked to answer the following questions on scales ranging from 1 (*not at all*) to 7 (*very much*): "Based on your experience and observations, to what extent do you think [outgroup members] feel the following emotions towards [ingroup members]: anger, disgust, contempt, respect (R)". The items were combined to form a reliable index of negative meta-emotions (α_{T1} = .84; α_{T2} = .90; α_{T3} = .85).

Meta-stereotypes. On scales ranging from 1 (not at all) to 7 (very much), participants answered the following questions: "Based on your experience and observations, to what extent do you think [outgroup members] rate [ingroup members] on the following traits: intelligent, intolerant". As the two items were only moderately correlated (r= -.27 at T1, r = -.36 at T2, r= -.20 at T3), we analysed them separately.

Attributions. We adapted six items (from Popan et al., 2010) to assess attributions about the sources of the outgroup's political views in terms of (ir-)rationality ("They have rational arguments to support their ideas"; "They do not seem to have put much thought or deliberation into their views" [R]), emotionality ("They hold their views because of their emotions", "They let their feelings guide their opinions"), and externality ("They hold their views because of the influence of their friends and family", "They hold their views because of the influence of media (TV, newspapers, etc.)" on 7-point scales (1 = *does not apply at all*, 7 = *applies very much*). The items were combined into separate indices of rationality (r_{T1} = .51; r_{T2} = .48; r_{T3} = .49, emotionality (r_{T1} = .85; r_{T2} = .89; r_{T3} = .82), and externality (r_{T1} = .38; r_{T2} = .72; r_{T3} = .53).

Contempt. Based on the theoretical literature on contempt (Fischer & Roseman, 2008) and work on the measurement of dispositional contempt (Schrijber et al., 2017), we generated items that assess the main cognitive appraisals and motivational goals of outgroup contempt in this context. Specifically, we assessed the appraisal of blameworthiness ("Leavers/Remainers are to blame for the current political problems in this country"), the perceived inferiority of the target of contempt (2 items, "I feel that Leavers/Remainers are inferior to me", "Leavers/Remainers have given me reasons to look down on them"), the feeling of a lack of control or influence over the target (2 items: "I think I can influence Leavers/Remainers"; "I think I can change Leavers'/Remainers' minds"), and the motivational goal of social rejection and avoidance (2 items: "In my personal life, I have wanted to break relationships as a result of the referendum"; I currently feel like I would go to some effort to avoid contact with Leavers/Remainers."). Using our full T1 sample (N=586), we conducted an exploratory factor analysis on these items with principal components extraction and oblimin rotation to explore the scale structure. We extracted two factors with Eigenvalues greater than one (3.37 and 1.49) that together explained 69.44% of the variance. Factor loadings showed that the first factor consisted of the items assessing blameworthiness, inferiority, and social rejection and the second factor contained the two items assessing perceived influence. Unexpectedly, and inconsistent with the literature (see Fischer & Roseman, 2008), the main contempt factor and perceived influence

were *positively* correlated (r=.18). We thus focused on the main contempt factor and discarded the items assessing perceived influence. Moreover, one item ("In my personal life, I have wanted to break all relationships as a result of the referendum") had relatively low communality (<.5) and consistently low (<.3) item-total correlations in the reliability analyses of the experimental data. This is likely to be due to its imprecise formulation. We thus decided to drop this item from further analysis. The four remaining items were combined into reliable indices of contempt (α_{T1} = .82; α_{T2} = .84; α_{T3} = .84).

Willingness to compromise. This was measured by indicating agreement with three items: "Compromising with [outgroup members] could lead to things that will enrich our country"; "If [outgroup members'] solution is proven to be better for the country, we should support them"; "We should try and find middle ground with [outgroup members] on issues where we can", which were adapted from Willis et al.'s (2017) Intergroup Compromise Inventory. Participants indicated their agreement with these statements on 7-point Likert scales ranging from 1 (*strongly disagree*) to 7 (*strongly agree*). The items were combined into a reliable measure of intergroup compromise willingness (α_{T1} = .71; α_{T2} = .80; α_{T3} = .81).

Collective action intentions. Participants were asked about how likely they are to engage in three actions (sign a petition, attend a demonstration, write a letter to the government) in order to protect the interests of Leavers/Remainer [INGROUP] on scales ranging from 1 (very unlikely) to 7 (very likely). The items were averaged into a composite index of collective action intentions (α_{T1} = .85, α_{T2} = .89, α_{T3} = .89).

Brexit opinions. We developed a new scale to assess participants' views regarding the main points of contention in the Brexit debate. On scales ranging from 1 (*strongly disagree*) to 7 (*strongly agree*), participants indicated their agreement with the following statements: "Brexit will allow the UK to take back control over immigration and its borders."; "Brexit will give the UK parliament more powers to shape British law"; "Brexit will protect British fishing rights"; "Stopping our payments to the EU budget will allow the UK to invest more money into the NHS"; "Brexit will allow the UK to increase its influence in the world"; "Brexit endangers stability in Europe and increases the risk of war"; "Brexit will have a negative impact on the economy, jobs, and prices"; "Brexit threatens worker's rights in this country"; "Brexit will make it more difficult to address global climate change"; "Brexit will make it more difficult to deal with terrorism in this country"; "Brexit threatens UK agriculture and food security". The items were presented in a random order. Principal components analysis using the full T1 data (*N* = 586) extracted a single factor (Eigenvalue = 7.64, variance explained = 69.4%). We therefore reversed pro-Brexit items and combined all items into a single composite with higher values indicating greater anti-Brexit views (α_{T1} = .95; α_{T2} = .94; α_{T3} = .95).

Intergroup anxiety. This measure was adapted from Stephan and Stephan (1985). On scales ranging from 1 (*not at all*) to 7 (*very much*), participants were asked to indicate the extent to which they would feel *anxious*, *comfortable* (R), *secure* (R), and *tense* if they were the only ingroup member interacting with a group of outgroup members. The items were reversed as appropriate and combined to form an index of intergroup anxiety (α_{T1} = .90; α_{T2} = .92; α_{T3} = .85).

Empathic concern for the outgroup. We adapted three items from the empathic concern subscale of Davis' (1983) interpersonal reactivity index for this context. On scales ranging from 1 (*strongly disagree*) to 7 (*strongly agree*), participants indicated their agreement with the following items: "If I knew that if a [outgroup member] was facing problems, I would feel sorry for them"; "Hearing of Leaver's misfortunes would not disturb

me a great deal" (R); "If I saw a Leaver being treated unfairly, I wouldn't feel much pity for them" (R). The items were reversed and combined to create an index of empathic concern for the outgroup (α_{T1} = .76; α_{T2} = .77; α_{T3} = .72).

Outgroup perspective taking. Three items assessing willingness to take the outgroup's perspective were adapted from the perspective taking subscale of Davis' (1983) interpersonal reactivity index for this context. Participants were asked to indicate their agreement with three items, e.g., "I sometimes find it difficult to see things from a [outgroups]'s point of view" [R]; "I sometimes try to understand [outgroup members] better by imagining how things look from their perspective"; "When facing political disagreements where I am sure I'm in the right, I believe listening to a [outgroup members'] arguments is a waste of my time" on scales ranging from 1 (*strongly disagree*) to 7 (*strongly agree*). Scale reliability analysis indicated that the three items did not form a reliable scale (α_{T1} = .40, α_{T2} = .53, α_{T3} = .49) due to the low item-total correlation of the reverse-coded item. We therefore dropped this item and combined the remaining two items into an index of outgroup perspective taking (r_{T1} = .35, p<.001; r_{T1} = .32, p=.014, for Leavers and .35, p = .007, for Remainers; r_{T2} = .31, p <.001; r_{T2} = .42, p<.001, for Leavers and .22, p=.09, for Remainers; r_{T3} = .33, p<.001; r_{T3} = .52, p < .001 for Leavers and .12, p = .36 for Remainers). Since the intercorrelations were still relatively low and non-significant at T2 and T3 for Remainers, we also analysed the items separately and report results in Tables S34-S36.

(5) Ancillary measures

The following measures were included at T1 and T3 only, for exploratory purposes:

Perceived similarity between Leavers and Remainers. At T1 and T3, participants indicated the extent to which the see similarities or differences between Remainers and Leavers in terms of: values, everyday concerns, and strength of community ties on scales ranging from 1 (*strong differences*) to 7 (*strong similarities*). The items were combined into reliable scales of perceived similarities (α_{T1} = .77; α_{T3} = .85).

Equality-based respect. Two items adapted from Renger and Reese (2017) were administered at T1 and T3 to assess experienced equality-based respect by the outgroup: "[Outgroup members] always communicate with [ingroup members] as with people of equal worth." "[ingroup members] are consistently treated by [outgroup members] as counterparts that are to be taken seriously." The items were combined into a composite score (r_{T1} = .62; r_{T3} = .74, all *ps* <.001).

Life satisfaction. Life satisfaction was measured at T1 and T3 with a single item adapted from the satisfaction with life scale (Diener, Lauren and Griffin, 1985) asking participants: "All things considered, how satisfied or dissatisfied are you with your life overall"? Responses were given on a 7-point Likert scale (1 = *very dissatisfied* to 7 = *very satisfied*).

Well-being. Mental wellbeing in the past two weeks was measured at T1 and T3 using the seven-item Short Warwick-Edinburgh Mental Wellbeing Scale (Ng et al., 2017). On 5-point Likert scales ranging from 1 (*none of the time*) to 5 (*all of the time*) participants responded to the following items: "I've been feeling optimistic about the future"; "I've been feeling useful"; "I've been feeling relaxed"; "I've been dealing with problems well"; "I've been thinking clearly"; "I've been feeling close to other people"; "I've been able to make up my own mind about things (α_{T1} = .90, α_{T3} = .90).

Supplementary Notes

Supplementary Notes 1

Timing of study and relevance of Brexit identities. It should be noted that there is polarization both in terms political party support and Brexit position in the United Kingdom. Evidence points to the differential importance of these identities as a function of time since the 2016 referendum, the substantive issue of concern (e.g., opinions on immigration), and what exactly is being measured. For example, using a range of data sets collected between 2016 and 2019, Hobolt et al. (2021) found that Brexit identities produced affective polarization (measured in terms of stereotypes, evaluative biases etc.) that was of equal or larger intensity than that of partisanship, and that the effects of the Brexit divide cut across traditional partisanship, highlighting the importance of this divide as a source of polarization over and above partisanship. Survey data collected during the 2019 general election campaigns also showed that identification with a Brexit side was stronger than identification with a political party, with both identities having gained in strength compared to 2018 (although this has been even more so for Brexit identities; The Policy Institute King's College London, 2019). When considering polarization around the issue of immigration in 2019, the divide on restricting immigration (a key factor for voting in favour of Brexit) was larger between Remainers and Leavers (66% disagree vs. 90% agree) than between supporters of Labour vs the Conservatives (51% disagree vs. 81% agree). The British Social Attitudes survey found no evidence that political polarization between Leavers and Remainers was decreasing in 2020/21 (see also Butler, 2021). Moreover, providing evidence for continuing polarization, 2020 saw a reversal of typical patterns of political trust, with Leavers more likely than Remainers to trust the government (NatCen, 2020). However, it should be noted that other evidence highlights that partisan identities can produce greater divisions than Brexit identities (Pew Research Center, 2019; Prike et al., 2023). Nonetheless, the key point is that the political identities that were the focus of our research were highly relevant and produced polarization in the UK population at the time that our research was conducted (between December 2020 until April 2021), something that was also evident in our data.

While Brexit remains a relevant issue in 2024 (National Centre for Social Research, 2024), more recently (from about 2022) there has indeed been a greater divide in terms of partisan identities and a decline in the importance of Brexit identities. Survey data suggests that a majority of Britons feel that the UK was wrong to leave the EU (56% to 32%), and that a sizeable proportion of Leave voters are regretting their vote (YouGov, 2022; The Policy Institute King's College London, 2022). Moreover, several recent scandals such as the violations of Covid restrictions ("partygate") revealed in the Sue Gray report published in May 2022, as well as the economic shock following Liz Truss' "mini-budget" in 2022, has brought partisan identities to the fore (e.g., British Politics at Queens, 2024).

Supplementary Results

Supplementary Results 1

Differences between Leavers and Remainers at T1 (Full sample, N = 586). Supplementary Table 4 summarizes means, standard deviations, effect sizes, and results of independent sample t-tests comparing Leavers and Remainers in the full T1 sample on demographics and study variables assessed at time 1.

Supplementary Table 4. Means (standard deviations), effect sizes, and significance tests for differences between Leavers (*N* = 192) and Remainers (*N* = 394) in the full T1 sample (*N* = 586)

Measure	Range	Group		Cohen's *d*	*t*-value[&]
		**Leaver**	**Remainer**		
Age	19-79	45.41 (13.59)	43.88 (14.10)	.11	1.25
Education	1-7	5.45 (1.07)	5.96 (0.99)	-.51	-5.58***
Identification	1-7	4.59 (1.24)	5.23 (1.13)	-.54	-6.19***
Contact (networks)	1-5	2.68 (1.02)	2.06 (.85)	.91	.42***
Contact (frequency)	1-5	3.51 (1.07)	2.98 (1.03)	.51	7.09***
Contact quality	1-7	6.04 (1.11)	5.13 (1.45)	.68	8.37***
Thermometer	0-100	55.48 (19.25)	34.29 (21.20)	1.03	11.96***
Negative outgroup emotions	1-7	2.57 (1.17)	3.98 (1.43)	-1.05	-12.76***
Stereotype: Intolerant	1-7	4.21 (1.88)	5.02 (1.64)	-.47	-5.10***
Stereotype: Intelligent	1-7	4.75 (1.25)	3.45 (1.40)	.96	11.37***
Meta-emotions	1-7	4.94 (1.45)	4.69 (1.36)	.18	2.01*
Meta: Intolerant	1-7	4.93 (1.73)	4.23 (1.74)	.41	4.59***
Meta: Intelligent	1-7	3.13 (1.64)	3.58 (1.39)	-.31	-3.27**
Rationality	1-7	4.58 (1.22)	3.15 (1.28)	1.14	12.93***
Emotionality	1-7	4.73 (1.42)	5.58 (1.30)	-.63	-7.12***
Externality	1-7	4.91 (1.27)	5.49 (1.12)	-.50	-5.46***
Contempt	1-7	2.48 (1.18)	3.82 (1.41)	-1.00	-12.06***
Compromise	1-7	4.87 (1.19)	4.74 (1.22)	.11	1.19
Collective action	1-7	3.70 (1.64)	4.90 (1.79)	-.69	-8.06***
Anti-Brexit views	1-7	1.41 (0.79)	4.34 (0.99)	-3.16	-38.64***
Intergroup anxiety	1-7	3.77 (1.62)	4.09 (1.51)	-.21	-2.36*
Empathic concern	1-7	5.49 (1.24)	5.15 (1.28)	.27	3.05***
Perspective taking	1-7	4.45 (1.33)	3.67 (1.40)	.57	6.44***

*Note. *** p <.001; **p <.01; * p <.05;* [&] degrees of freedom vary from 327.78 to 584 due to correction.

Supplementary Results 2

Detailed Reporting of Tests for Selective Attrition. We compared T1 participants who indicated that they would like to continue with the study (i.e., to participate in the interaction; N= 484) with participants who indicated that they would not like to participate further (N=102) in terms of demographic and attitudinal variables (see Supplementary Tables 5a and 6a for results). These analyses indicated that while men and women were equally likely to drop out, Remainers were more likely to drop out than Leavers, as were older compared to younger people, and participants who reported less frequent outgroup contact and lower contact quality. In addition, continuers and drop-outs differed significantly in terms of a number of attitudinal variables. Specifically, those who decided to leave the study were more strongly identified with their group, had more extreme views about Brexit, rated the outgroup as less intelligent, perceived the outgroup's views as less rational and more strongly based on external factors, reported higher levels of contempt and lower levels of warmth, perspective taking, and empathic concern, and were less willing to compromise and more willing to engage in collective action on behalf of their ingroup. There were significant differences between Remainers and Leavers in terms of the role of meta-perceptions (intelligent) and meta-emotions in predicting drop-out. While Leavers who thought the outgroup viewed them as less intelligent were more likely to continue with the study, the opposite was true for Remainers. Moreover, negative meta-emotions favoured drop-out for Remainers but not for Leavers.

Supplementary Table 5a. Differences between Stage 1 dropouts and continuers (dichotomous variables)

	**Dropout**	**Continuer**	***χ2***
Gender	Female = 47 (46.1%)<br>Male = 55 (53.9%)	Female = 253 (52.3%)<br>Male = 226 (46.7%)	2.60<br>(*p* =.626)
Group	Leaver = 23 (22.5%)<br>Remainer = 79 (77.5%)	Leaver = 169 (34.9%)<br>Remainer = 315 (65.1%)	5.85*<br>(*p* =.016)

Supplementary Table 6a. Differences between Stage 1 dropouts and continuers, by group (continuous variables)

	Leavers				Remainers				Whole sample			
	Dropout (N=23)	Continuer (N=169)	*d*	t-value	Dropout (N=79)	Continuer (N=315)	*d*	t-value	Dropout (N=102)	Continuer (N=484)	d	t-value
**Age**	50.7	44.69	.45	1.66^{+}	47.29	42.91	.31	2.28*	48.07	42.53	.33	2.69**
**Education**	5.43	5.55	-.01	-.06	6.13	5.92	.21	1.65^{+}	5.97	5.76	.21	2.00^{+}
**Identification**	5.04	4.53	.41	1.86^{+}	5.34	5.20	.12	.95	5.27	4.97	.25	2.32*
**Contact (network)**	2.64	2.69	-.04	-.19	1.98	2.08	-.12	-.93	2.14	2.29	-.16	-1.47
**Contact (overall)**	3.26	3.54	-.27	-1.20	2.81	3.03	-.21	-1.66^{+}	2.91	3.21	-.28	-2.53*
**Contact quality**	5.95	6.05	-.10	-.44	4.76	5.22	-.21	-2.53*	5.03	5.51	-.34	-2.96**
**Thermometer**	47.00	56.61	-.50	-2.22*	29.19	35.59	-.30	-2.39*	33.11	42.96	-.44	-3.97***
**Negative outgroup emotions**	2.58	2.56	.01	.05	4.31	3.90	.29	2.25*	3.91	3.44	.32	2.88**
**Stereotype: Intolerant**	4.26	4.20	.03	.15	5.09	5.00	.05	.42	4.90	4.72	.10	.92
**Stereotype: Intelligent**	4.78	4.75	.03	.12	3.05	3.55	-.36	-2.82**	3.45	3.97	-.35	-3.23**
**Meta-emotions[§]**	4.64	4.98	-.23	-1.05	5.08	4.60	.35	2.80**	4.98	4.73	.18	1.61
**Meta: Intolerant**	5.00	4.92	.05	.20	4.39	4.19	.11	.88	4.53	4.45	.05	.43
**Meta: Intelligent[§]**	3.83	3.03	.49	2.20*	3.08	3.70	-.45	-3.51***	3.26	3.46	-.14	-1.26
**Rationality**	4.52	4.59	-.06	-.26	2.91	3.20	-.23	-1.81^{+}	3.28	3.69	-.29	-2.64**
**Emotionality**	4.61	4.75	-.10	-.45	5.70	5.44	.12	.95	5.45	5.27	.13	1.19
**Externality**	5.24	4.86	.30	1.34^{+}	5.58	5.47	.10	.78	5.51	5.26	.20	1.99*

Supplementary Table 6a. Continued.

	Leavers				Remainers				Whole sample			
	Dropout (N=23)	Continuer (N=169)	*d*	t-value	Dropout (N=79)	Continuer (N=315)	*d*	t-value	Dropout (N=102)	Continuer (N=484)	d	t-value
**Contempt**	2.96	2.51	.37	1.82⁺	4.16	3.80	.26	1.96⁺	3.83	3.29	.37	3.38***
**Compromise**	4.54	4.91	-.32	-1.44	4.45	4.82	-.31	-2.43*	4.47	4.85	-.32	-2.93**
**Collective action**	3.88	3.67	.13	.58	5.12	4.84	.16	1.23	4.84	4.44	.22	2.03*
**Anti-Brexit views**§	1.10	1.45	-.44	-2.00*	4.54	4.30	.25	1.95⁺	3.76	3.30	.28	2.56*
**Intergroup anxiety**	3.57	3.80	-.14	-.63	4.21	4.06	.10	.81	4.07	3.97	.06	.59
**Empathic concern**	5.30	5.52	-.17	-.77	4.85	5.22	-.29	-2.32*	4.95	5.33	-.29	-2.68**
**Perspective taking**	4.11	4.50	-.27	-1.33	3.55	3.70	-.11	-.87	3.68	3.98	-.21	-1.97*

*Note. *** p <.001; **p <.01; * p <.05; + p <.10,* § denotes a significant interaction between continuation and group (Leaver, Remainer)

Next, we compared participants who declared at T1 that they would like to participate in the interaction but failed to respond or show (N= 262) with participants who participated in the interaction (N=120) in terms of T1 demographic and attitudinal variables (see Tables S5b and S6b for results). Note that we did not include participants who agreed to participate but could not be assigned a partner (N=102) as non-participation was due to external factors. All of these participants were Remainers who could not be assigned a partner due to the fact that many more Remainers than Leavers entered the study.

Supplementary Table 5b. Differences between no-shows and completers (dichotomous variables)

	**No show**	**Completer**	***χ2***
Gender	Female = 151<br>Male = 109	Female = 67<br>Male = 53	.17<br>(*p* =.738)
Group	Leaver = 109<br>Remainer = 153	Leaver = 60<br>Remainer = 60	2.35<br>(*p* =.149)

Supplementary Table 6b. Differences between no-shows and completers, by group (continuous variables)

	Leavers				Remainers				Whole sample			
	No-show (N=109)	Completer (N=60)	*d*	t-value	No-show (N=153)	Completer (N=60)	*d*	t-value	No-show (N=262)	Completer (N=120)	d	t-value
**Age**	43.49	46.85	-.26	-1.61	37.15	42.30	-.47	-2.57**	39.78	44.58	-.39	-3.34***
**Education**	5.47	5.42	.05	.30	5.75	6.05	-.30	-2.19^{+}	5.63	5.73	-.10	-.86
**Identification**	4.57	4.47	.09	.53	4.90	4.82	.07	.48	4.76	4.65	.10	.94
**Contact (network)**	2.67	2.72	-.05	-.29	2.16	1.85	.38	2.64*	2.36	2.29	.08	.72
**Contact (overall)**	3.54	3.55	-.01	-.05	3.08	3.03	.04	.27	3.27	3.29	-.02	-.18
**Contact quality**	6.03	6.10	-.06	-.37	5.72	5.64	.07	-.46	5.85	5.87	-.02	-.13
**Thermometer**	57.06	55.82	.07	.41	40.90	40.64	.01	.08	47.62	48.29	-.03	-.29
**Negative outgroup emotions**	2.65	2.41	.20	1.32	3.53	3.43	.08	.52	3.17	2.92	.19	1.68^{+}
**Stereotype: Intolerant**	4.11	4.35	-.12	-.77	4.75	4.88	-.09	-.58	4.48	4.62	-.08	-.68
**Stereotype: Intelligent**	4.59	5.03	-.36	-2.27*	3.90	3.90	.00	.01	4.19	4.47	-.21	-1.89^{+}
**Meta-emotions**	5.00	4.95	.03	.18	4.26	4.57	-.24	-1.55	4.57	4.76	-.14	1.26
**Meta: Intolerant**	4.92	4.93	-.01	-.06	3.89	4.40	-.31	-2.01*	4.32	4.67	-.20	-1.81^{+}
**Meta: Intelligent**	3.06	2.98	.05	.28	3.82	3.66	.13	.86	3.50	3.32	.13	1.15
**Rationality**	4.72	4.37	-29	1.77^{+}	3.46	3.50	-.04	-.23	3.98	3.93	.04	.32
**Emotionality**	4.81	4.65	.11	.68	5.27	5.38	-.09	-.62	5.08	5.02	.05	.42
**Externality**	4.84	4.90	-.05	-.33	5.36	5.35	.01	.05	5.14	5.13	.02	.13

Supplementary Table 6b. Continued.

	Leavers				Remainers				Whole sample			
	No-show (N=109)	Completer (N=60)	*d*	t-value	No-show (N=153)	Completer (N=60)	*d*	t-value	No-show (N=262)	Completer (N=120)	*d*	t-value
**Contempt**	2.47	2.33	.12	.74	3.29	3.27	.02	.12	2.96	2.80	.12	1.05
**Compromise**	4.82	5.08	-.22	-1.36	5.04	5.28	-.26	$-1.68^{=}$	4.94	5.18	-.22	-1.99*
**Collective action**	3.68	3.66	.01	.06	4.29	4.01	.16	.98	4.03	3.83	.12	1.07
**Anti-Brexit views**	1.43	1.48	-.07	-.41	3.92	3.94	-.03	-.17	2.89	2.71	.12	1.05
**Intergroup anxiety**	3.92	3.58	.21	1.30	3.97	4.04	-.05	-.32	3.95	3.81	.09	.81
**Empathic concern**	5.60	5.37	.19	-1.15	5.45	5.42	.03	.17	5.51	5.39	.10	.88
**Perspective taking**	4.42	4.65	-.18	-1.13	3.86	4.08	-.16	-1.06	4.09	4.37	-.20	-1.83^{+}

Note. **** p <.001; **p <.01; * p <.05; + p <.10.* There were no significant interactions between completion and group (Leaver, Remainer)

The analyses indicated that dropout at the later stages of the experiment, though non-response to correspondence to schedule an interaction or failure to show for a scheduled interaction, was linked to two variables. Specifically, younger people and those less willing to compromise with the outgroup were more likely to drop out. There were no significant differences between Leavers and Remainers. We can only speculate why these variables might have contributed to dropout at this stage. Participation in the interaction required participants to find a quiet space and period of uninterrupted time. It is plausible that younger participants might have more conflicting commitments and demands on their time, for example due to caring responsibilities for young children, which might have impacted on their ability to complete the study. Regarding the (lack of) willingness to compromise as a factor that may have led to dropout, we think it is possible that those who were less willing to compromise with the outgroup might have been put off by the ground rules for the interaction, which we laid out in our communications prior to the interaction. It is possible that participants who were less willing to compromise would have preferred a more confrontational interaction style and were less interested in engaging in dialogue.

Supplementary Results 3

Group differences at T1 (Intervention sample, N = 120). Supplementary Table 7 summarizes means, standard deviations, effect sizes, and results of independent sample t-tests comparing Leavers and Remainers in the intervention sample on demographics and study variables assessed at T1.

Supplementary Table 7. Means (standard deviations), effect sizes, and significance tests for differences between Leavers (*N* = 60) and Remainers (*N* = 60) in the intervention sample at T1

		Group			
Measure	Range	**Leaver**	**Remainer**	**Cohen's *d***	***t*-value[&]**
Age	24-79	46.85 (13.35)	42.92 (12.84)	-.30	-1.64
Education	1-7	5.42 (1.08)	6.05 (.81)	.66	3.64***
Identification	1-7	4.47 (1.11)	4.83 (1.23)	.30	1.66[+]
Contact (networks)	1-5	2.66 (1.03)	1.18 (.72)	-.97	-5.30***
Contact (overall)	1-5	3.55 (1.03)	3.03 (1.04)	-.50	-2.73**
Contact quality	1-7	6.10 (1.10)	5.64 (1.13)	-.41	-2.25*
Thermometer	0-100	55.82 (17.69)	40.64 (21.89)	-.76	-4.16***
Negative outgroup emotions	1-7	2.41 (1.01)	3.43 (1.33)	.86	4.71***
Stereotype: Intolerant	1-7	4.35 (1.82)	4.88 (1.46)	.32	1.77[+]
Stereotype: Intelligent	1-7	5.03 (1.16)	3.90 (1.26)	-.94	-5.12***
Negative meta-emotions	1-7	4.95 (1.35)	4.57 (1.31)	-.29	-1.60
Meta: Intolerant	1-7	4.93 (1.59)	4.40 (1.84)	-.31	-1.70[+]
Meta: Intelligent	1-7	2.98 (1.58)	3.66 (1.20)	.48	2.64*
Rationality	1-7	4.37 (1.37)	3.50 (1.18)	-.68	-3.71***
Emotionality	1-7	4.65 (1.50)	5.38 (1.23)	.54	2.93**
Externality	1-7	4.90 (1.08)	5.35 (1.15)	.40	2.22*
Contempt	1-7	2.34 (.98)	3.31 (1.31)	.84	4.58***
Compromise	1-7	5.08 (1.14)	5.28 (.86)	.20	1.12
Collective action	1-7	3.66 (1.59)	4.01 (1.95)	.19	1.06
Anti-Brexit views	1-7	2.48 (.81)	4.94 (1.00)	2.70	14.80***
Intergroup anxiety	1-7	3.58 (1.63)	4.07 (1.09)	.36	1.95[+]
Empathic concern	1-7	5.37 (1.26)	5.42 (1.13)	.04	.20
Perspective taking	1-7	4.65 (1.18)	4.08 (1.36)	-.45	-2.44*

Note. *** *p* <.001; ***p* <.01; * *p* <.05; + *p* <.10; [&] degrees of freedom vary from 118 to 105.44 due to correction.

Supplementary Results 4

Supplementary Table 8 gives an overview of mean ratings and standard deviations for ratings of the interaction as a function of group and condition.

Supplementary Table 8. Ratings of the interaction by group and condition.

Condition	Intervention			Control		
Group	Leaver	Remainer	All	Leaver	Remainer	All
Positive affect	3.73 (.77)	3.26 (.73)	3.50 (.78)	3.59 (.89)	3.32 (1.02)	3.46 (.96)
Negative affect	1.06 (.19)	1.15 (.29)	1.10 (.25)	1.13 (.30)	1.18 (.47)	1.16 (.39)
Quality	6.73 (.53)	6.56 (.80)	6.65 (.68)	6.83 (.32)	6.72 (.38)	6.78 (.35)
Equality-based respect	6.81 (.38)	6.74 (.54)	6.77 (.47)	6.82 (.37)	6.80 (.47)	6.81 (.42)
Perceived similarity	5.70 (1.01)	5.06 (1.13)	5.38 (1.11)	5.98 (.95)	5.60 (1.04)	5.78 (1.01)

Note. N = 62 (31 Leavers, 31 Remainers) in the Intervention condition and *N* = 58 (29 Leavers, 29 Remainers) in the Control condition. All variables were assessed using 7-point Likert-type scales.

We assessed the effect of condition (control vs. intervention), group (Leaver vs. Remainer) as well as their interaction. We employed multi-level analyses (Hox et al., 2018) using Mplus (Muthén & Muthén, 1998–2017) for these tests to account for nesting of individuals (level 1) within dyads (level 2), specifying group as a level 1 factor and condition as a level 2 factor. As perceived quality of the interaction and perceived equality-based respect during the interaction were highly positively skewed, we repeated the analysis with dichotomized variables for these variables. There were no significant effects of condition or group, nor were there significant interactions between group and condition, for positive and negative state affect, quality of the interaction, equality-based respect during the interaction, or perceived similarity of the interaction partner. For detailed results, see Supplementary Tables 9-15 below. Overall, these findings indicate that the control and intervention conditions were experienced similarly by the participants and that any experimental effects can therefore not be attributed to the experience of a positive interaction per se.

Supplementary Table 9. Full results of comparisons of intervention and control interaction for <u>positive state affect</u>.

	Model 1		Model 2		Model 3		Model 4	
	B	SE	B	SE	B	SE	B	SE
Intercept	3.48***	0.09	3.27***	0.13	3.27***	0.13	3.32***	0.14
Group (0 = R 1 = L)			0.37*	0.15	0.37*	0.16	0.28	0.21
Condition (0 = C 1 = E)			0.04	0.17	0.04	0.18	-0.05	0.23
Group × Condition							0.19	0.32
Variance DV L1	0.74***	0.14	0.70***	0.16	0.70***	0.18	0.69***	0.18
Variance DV L2	0.01	0.11	0.01	0.12	0.01	0.12	0.01	0.12
Variance Group random T L2					0.02	0.20	0.02	0.20
AIC	311.315		309.481		311.536		313.159	
BIC	319.677		323.418		328.261		332.671	

Note. $ICC_{Level\ 2}$ = 0.009. ****p* <.001; ***p* < .01, *, *p* <.05, †*p* <.10.

Supplementary Table 10. Full results of comparisons of intervention and control interaction for <u>negative state affect.</u>

	Model 1		Model 2		Model 3		Model 4	
	B	SE	B	SE	B	SE	B	SE
Intercept	1.13***	0.07	1.19***	0.09	1.19***	0.09	1.18***	0.10
Group (0 = R 1 = L)			-0.08	0.08	-0.08	0.16	-0.05	0.17
Condition (0 = C 1 = E)			-0.05	0.08	-0.05	0.08	-0.03	0.09
Group × Condition							-0.05	0.14
Variance DV L1	0.10†	0.05	0.10†	0.05	0.10†	0.05	0.10	0.05
Variance DV L2	0.00	0.06	0.00	0.06	0.00	0.06	0.00	0.06
Variance Group random T L2					0.00	0.03	0.00	0.03
AIC	73.970		75.572		77.599		79.448	
BIC	82.332		89.510		94.324		98.960	

Note. $ICC_{Level\ 2}$ = 0.008. ****p* <.001; ***p* < .01, *, *p* <.05, †*p* <.10.

Supplementary Table 11. Full results of comparisons of intervention and control interaction for <u>quality of the interaction</u>.

	Model 1		Model 2		Model 3		Model 4	
	B	SE	B	SE	B	SE	B	SE
Intercept	6.71***	0.12	6.71***	0.16	6.71***	0.16	6.72***	0.17
Group (0 = R 1 = L)			0.14	0.10	0.14	0.14	0.11	0.19
Condition (0 = C 1 = E)			-0.13	0.18	-0.13	0.19	-0.16	0.20
Group × Condition							0.06	0.20
Variance DV L1	0.21***	0.02	0.20***	0.02	0.20***	0.02	0.20***	0.03
Variance DV L2	0.09**	0.03	0.09**	0.03	0.09**	0.03	0.09	0.03
Variance Group random T L2					0.001	0.09	0.001	0.09
AIC	195.997		195.711		197.816		199.692	
BIC	204.359		209.648		214.541		219.204	

Note. $ICC_{Level\ 2}$ = 0.305. ****p* <.001; ***p* < .01, *, *p* <.05, †*p* <.10.

Supplementary Table 12. Full results of comparisons of intervention and control interaction for <u>quality of the interaction (dichotomized).</u>

	Model 1		Model 2		Model 3		Model 4	
	B	SE	B	SE	B	SE	B	SE
Threshold	-0.26	0.21	0.36	0.39	0.64	2.99	0.41	39.00
Group (0 = R 1 = L)			1.05*	0.44	3.81	39.72	1.25	530.13
Condition (0 = C 1 = E)			0.23	0.47	0.52	2.99	0.34	32.16
Group × Condition							-0.21	42.44
Variance DV L1								
Variance DV L2	0.42	0.74	0.82	0.98	5.58	86.13	0.92	615.94
Variance Group random T L2					54.58	1320.60	0.74	4222.37
AIC	168.258		165.493		167.424		169.423	
BIC	173.833		176.643		181.361		186.148	

Note. $ICC_{Level\ 2}$ = 0.114. ****p* <.001; ***p* < .01, *, *p* <.05, †*p* <.10.

Supplementary Table 13. Full results of comparisons of intervention and control interaction for <u>equality-based respect during the interaction</u>.

	Model 1		Model 2		Model 3		Model 4	
	B	SE	B	SE	B	SE	B	SE
Intercept	6.79***	0.10	6.79***	0.13	6.79***	0.14	6.81***	0.15
Group (0 = R 1 = L)			0.04	0.08	0.04	0.15	0.01	0.18
Condition (0 = C 1 = E)			-0.04	0.11	-0.04	0.11	-0.07	0.13
Group × Condition							0.06	0.16
Variance DV L1	0.16***	0.04	0.15***	0.04	0.15**	0.06	0.15**	0.06
Variance DV L2	0.04	0.02	0.04	0.03	0.04	0.04	0.04	0.04
Variance Group random T L2					0.001	0.08	0.001	0.08
AIC	146.807		150.253		152.344		154.145	
BIC	155.169		164.190		169.069		173.658	

Note. $ICC_{Level\ 2}$ = 0.203. ****p* <.001; ***p* < .01, *, *p* <.05, †*p* <.10.

Supplementary Table 14. Full results of comparisons of intervention and control interaction for <u>equality-based respect during the interaction (dichotomized).</u>

	Model 1		Model 2		Model 3		Model 4	
	B	SE	B	SE	B	SE	B	SE
Threshold	-1.30***	0.35	-1.47**	0.54	-1.52	0.85	-1.70	53.96
Group (0 = R 1 = L)			-0.32	0.47	-0.17	250.67	-0.54	108.29
Condition (0 = C 1 = E)			0.02	0.57	-0.01	0.97	-0.33	9.45
Group × Condition							0.70	60.12
Variance DV L1								
Variance DV L2	1.22	1.29	1.27	1.35	1.50	450.89	1.55	261.23
Variance Group random T L2					1.03	2112.68	1.02	1182.23
AIC	139.397		142.920		144.922		146.411	
BIC	144.972		154.070		158.859		163.136	

Note. $ICC_{Level\ 2}$ = 0.271. ****p* <.001; ***p* < .01, *, *p* <.05, †*p* <.10.

Supplementary Table 15. Full results of comparisons of intervention and control interaction for <u>perceived similarity of interaction partner</u>.

	Model 1		Model 2		Model 3		Model 4	
	B	SE	B	SE	B	SE	B	SE
Intercept	2.42***	0.13	2.47***	0.18	2.47***	0.20	2.40***	0.21
Group (0 = R 1 = L)			-0.51**	0.16	-0.51**	0.16	-0.38	0.26
Condition (0 = C 1 = E)			0.41†	0.22	0.41†	0.25	0.53†	0.28
Group × Condition							-0.26	0.34
Variance DV L1	0.84***	0.16	0.71***	0.15	0.70***	0.18	0.69***	0.18
Variance DV L2	0.31*	0.15	0.34*	0.15	0.34*	0.15	0.34*	0.15
Variance Group random T L2					0.02	0.36	0.02	0.36
AIC	358.924		349.322		351.459		352.768	
BIC	367.287		363.260		368.184		372.281	

Note. $ICC_{Level\ 2}$ = 0.272. ****p* <.001; ***p* < .01, *, *p* <.05, †*p* <.10.

Supplementary Results 5

Supplementary Table 16 presents means and standard deviations of focal outcome variables by condition, group, and measurement point.

Supplementary Table 16. Means and standard deviations of focal outcome variables by condition, group, and measurement point

	Intervention						Control					
	Remainer			Leaver			Remainer			Leaver		
Measures	T1	T2	T3	T1	T2	T3	T1	T2	T3	T1	T2	T3
Thermometer	36.58 (19.87)	51.29 (20.47)	44.52 (18.67)	55.74 (15.53)	65.61 (17.37)	59.58 (17.89)	45.14 (23.48)	43.71 (20.63)	44.57 (23.06)	55.90 (20.01)	57.10 (18.33)	57.43 (18.97)
Negative emotions	3.58 (1.29)	2.92 (1.18)	2.90 (1.06)	2.30 (1.05)	1.89 (0.76)	1.92 (0.80)	3.27 (1.37)	3.13 (1.25)	3.14 (1.40)	2.53 (0.97)	2.31 (0.89)	2.15 (0.78)
Stereotype: Intolerant	4.94 (1.53)	3.65 (1.54)	4.19 (1.35)	3.93 (1.73)	3.30 (1.94)	3.81 (1.80)	4.83 (1.42)	4.07 (1.67)	4.59 (1.64)	4.70 (1.85)	4.06 (1.82)	3.94 (1.69)
Stereotype: Intelligent	3.94 (1.15)	4.87 (1.09)	4.63 (0.96)	4.97 (1.28)	5.19 (1.58)	5.55 (1.03)	3.86 (1.38)	3.75 (1.46)	4.43 (1.50)	5.10 (1.05)	5.07 (1.25)	5.00 (1.09)
Negative meta-emotions	4.56 (1.13)	3.64 (1.42)	3.85 (1.12)	4.91 (1.32)	4.37 (1.67)	4.17 (1.64)	4.58 (1.49)	4.28 (1.36)	3.96 (1.32)	5.00 (1.40)	4.52 (1.80)	4.42 (1.44)
Meta-intolerant	4.94 (1.83)	3.97 (1.58)	3.90 (1.45)	5.10 (1.66)	4.23 (2.05)	4.48 (2.11)	3.83 (1.71)	3.96 (1.71)	4.45 (1.81)	4.76 (1.53)	4.83 (1.51)	4.61 (1.75)
Meta-intelligent	3.53 (1.17)	4.26 (1.37)	4.35 (1.31)	2.94 (1.59)	3.45 (1.61)	3.23 (1.54)	3.79 (1.24)	3.71 (1.38)	4.32 (1.47)	3.03 (1.59)	3.31 (1.65)	3.96 (1.73)
Rationality	3.39 (0.95)	4.60 (1.23)	3.97 (1.09)	4.29 (1.45)	4.89 (1.36)	4.90 (1.39)	3.62 (1.39)	3.80 (1.12)	3.64 (1.21)	4.45 (1.30)	4.93 (1.12)	4.59 (1.03)
Emotionality	5.53 (1.13)	4.60 (1.23)	5.48 (0.99)	5.09 (1.13)	4.07 (1.79)	4.89 (1.35)	5.22 (1.32)	5.21 (1.34)	5.32 (1.34)	4.50 (1.61)	4.14 (1.46)	4.43 (1.36)
Externality	5.58 (1.03)	4.52 (1.61)	5.40 (1.16)	4.85 (1.13)	4.19 (1.65)	4.66 (1.33)	5.10 (1.23)	5.30 (1.02)	5.38 (1.09)	4.95 (1.03)	4.53 (1.36)	4.93 (1.09)
Contempt	3.40 (1.21)	2.49 (1.23)	3.10 (1.02)	2.29 (.94)	1.93 (1.07)	2.18 (1.11)	3.21 (1.44)	2.97 (1.53)	3.12 (1.52)	2.39 (1.03)	2.16 (.89)	2.22 (.93)

Supplementary Table 16. ***Continued.***

	Intervention						Control					
	Remainer			Leaver			Remainer			Leaver		
**Measures**	**T1**	**T2**	**T3**	**T1**	**T2**	**T3**	**T1**	**T2**	**T3**	**T1**	**T2**	**T3**
Compromise willingness	5.13 (0.85)	5.87 (0.82)	5.73 (0.74)	5.05 (1.27)	5.56 (1.23)	5.13 (1.50)	5.45 (0.85)	5.56 (1.09)	5.49 (1.01)	5.10 (1.00)	5.26 (1.00)	5.20 (1.09)
Collective action	4.25 (1.82)	4.27 (1.90)	4.18 (1.86)	3.57 (1.57)	2.83 (1.54)	3.11 (1.68)	3.74 (2.08)	3.45 (2.19)	3.57 (2.23)	3.86 (1.61)	3.71 (1.90)	3.50 (1.81)
Ant-Brexit Opinions	4.93 (0.91)	4.32 (1.00)	4.53 (0.99)	2.50 (0.74)	2.54 (0.88)	2.55 (0.93)	4.96 (1.11)	4.86 (1.13)	4.86 (1.27)	2.46 (0.89)	2.41 (0.82)	2.52 (0.96)
Intergroup anxiety	4.34 (1.08)	3.95 (1.13)	3.99 (0.95)	3.37 (1.77)	3.61 (1.87)	3.31 (1.72)	3.78 (1.04)	3.91 (1.11)	3.92 (1.25)	3.80 (1.46)	3.66 (1.79)	3.63 (1.61)
Empathic concern	5.47 (1.04)	5.91 (0.96)	5.80 (1.06)	5.33 (1.40)	6.06 (1.03)	5.62 (1.03)	5.36 (1.23)	5.42 (1.14)	5.30 (1.10)	5.41 (1.11)	5.75 (1.05)	5.45 (1.12)
Perspective taking	3.85 (1.40)	4.44 (1.33)	4.31 (1.01)	4.69 (1.12)	4.98 (1.50)	4.73 (1.57)	4.33 (1.29)	4.34 (1.18)	4.31 (1.01)	4.60 (1.26)	4.76 (1.17)	4.59 (1.25)

Note. Outgroup warmth is measured on a feeling thermometer ranging from 0 to 100. All other variables are measured on 7-point scales, with higher values indicating greater agreement or willingness.

Supplementary Results 6

To assess the short- and longer-term effects of our manipulation on the focal outcomes, we employed multi-level analyses (Hox et al., 2018) using Mplus (Muthén & Muthén, 1998–2017) We tested for experimental effects in a series of three-level models in Mplus, accounting for nesting of measurement points (level 1) within individuals (level 2) and nesting of individuals within dyads (level 3). In each of these models, time was coded with a dummy variable comparing measurement at T1 with measurement at T2 (to assess short-term effects) or measurement at T1 with measurement at T3 (to assess longer-term effects). Changes over time are represented by the effects of dummy variables capturing short-term (T1-T2) and longer-term (T1-T3) effects, respectively. Experimental effects are indicated by a significant cross-level interaction between condition (0 = control group, 1 = intervention group), which was specified as a level-3 variable, and the slope representing time. We also examined whether short- and longer-term effects were moderated by group membership by testing for three-way interactions between time, condition, and group (Leaver = 0 vs. Remainer = 1). Full details of the analyses and results are presented in Supplementary Tables 17-36 below.

In Model 1, the dummy variable was specified as a fixed predictor of a given outcome variable. In Model 2 (a random-effects model), the effect of time was allowed to differ between individuals and dyads. In other words, Model 2 includes two random slopes. In Model 3, condition (0 = control group, 1 = intervention group) – a level-3 variable – is specified to predict the outcome variable and the slope. The latter will test for a cross-level interaction – between condition and dummy variable capturing short-term effects (T1 vs. T2) or, to assess longer-term effects, between condition and the T1-T3 dummy variable. Significant interactions indicate that changes in the level of the outcome variable between T1 and T2 or T1 and T3, differ between conditions. To check if the pattern of results is consistent with our expectations, we probe the significant interactions.

Supplementary Table 17. Full results for outgroup warmth (feeling thermometer).

	Model 1		Model 2		Model 3		Model 4		Model 5	
	B	SE	B	SE	B	SE	B	SE	B	SE
Intercept	51.58***	1.89	40.64***	2.62	41.03***	2.63	45.11***	2.96	45.11***	3.34
T1 vs T2			6.42***	1.63	5.86**	1.84	0.61	3.22	-0.07	3.71
T1 vs T3			3.31†	1.72	3.02†	1.77	3.18†	1.67	3.19†	1.70
Group (0 = R 1 = L)			14.00***	3.07	13.92***	3.18	8.86†	4.98	8.71†	5.08
Condition (0 = C 1 = E)			1.35	3.28	1.30	3.21	-3.34	4.90	-3.59	5.23
Group × Condition							3.16	6.92	3.67	7.58
T1 vs T2 × Group							1.06	2.87	2.63	5.46
T1 vs T2 × Condition							9.61**	3.45	10.90*	4.76
T1 vs T2 × Group × Condition									-2.90	6.22
T1 vs T3 × Group										
T1 vs T3 × Condition										
T1 vs T3 × Group × Condition										
Variance DV L1	158.28***	9.79	148.00***	10.91	147.28***	19.01	139.05***	18.91	138.57***	18.81
Variance DV L2	270.88***	45.50	215.76***	44.54	220.61***	47.90	244.46***	61.80	244.63***	65.12
Variance DV L3	3.10	38.92	11.76	33.92	6.21	34.11	2.49	38.98	2.40	39.24
Variance random L2					1.08	61.44	4.07	60.39	4.12	70.29
Variance random L3					1.09	25.82	0.56	26.43	0.55	27.94
Variance group							0.49	87.04	0.48	92.64
AIC	3038.343		3010.487		3014.462		3011.664		3015.053	
BIC	3053.843		3041.486		3053.212		3069.788		3081.000	

Supplementary Table 17. ***Continued.***

	Model 6		Model 7		Model 8	
	B	SE	B	SE	B	SE
Intercept	40.95***	2.62	43.31***	3.12	43.35***	3.76
T1 vs T2	6.08***	1.72	6.27**	1.80	6.26**	1.82
T1 vs T3	2.63	1.92	3.00	4.05	2.68	5.27
Group (0 = R 1 = L)	14.00***	3.29	8.83	5.38	8.74	6.29
Condition (0 = C 1 = E)	1.39	3.22	-0.19	4.36	-0.29	4.83
Group × Condition			3.62	6.87	3.87	7.77
T1 vs T2 × Group						
T1 vs T2 × Condition						
T1 vs T2 × Group × Condition						
T1 vs T3 × Group			0.91	3.41	1.61	5.85
T1 vs T3 × Condition			-0.77	3.94	-0.16	5.98
T1 vs T3 × Group × Condition					-1.40	7.21
Variance DV L1	147.53***	14.59	147.60***	15.02	147.45***	15.06
Variance DV L2	213.19***	57.24	217.55***	63.91	217.60**	65.78
Variance DV L3	6.94	35.58	1.83	37.43	1.84	38.35
Variance random L2	1.28	49.78	1.18	50.97	1.17	64.06
Variance random L3	0.56	35.32	0.23	35.76	0.23	38.22
Variance group			0.48	86.54	0.48	86.57
AIC	3016.294		3024.703		3028.575	
BIC	3058.918		3082.827		3094.448	

Note. $ICC_{Level\ 3}$ = 0.007, $ICC_{Level\ 2}$ = 0.627. ****p* <.001; ***p* < .01, *, *p* <.05, †*p* <.10.

Supplementary Table 18. Full results for negative outgroup emotions.

	Model 1		Model 2		Model 3		Model 4		Model 5	
	B	SE	B	SE	B	SE	B	SE	B	SE
Intercept	2.67***	0.14	3.46***	0.16	3.46***	0.16	3.37***	0.18	3.36***	0.19
T1 vs T2			-0.36***	0.09	-0.36***	0.09	-0.32*	0.15	-0.29†	0.17
T1 vs T3			-0.40***	0.07	-0.40***	0.07	-0.40***	0.07	-0.40***	0.07
Group (0 = R 1 = L)			-0.93***	0.20	-0.92***	0.21	-0.81*	0.34	-0.79*	0.35
Condition (0 = C 1 = E)			-0.14	0.22	-0.16	0.22	0.04	0.26	0.07	0.27
Group × Condition							-0.27	0.44	-0.32	0.45
T1 vs T2 × Group							0.10	0.14	0.03	0.21
T1 vs T2 × Condition							-0.14	0.19	-0.21	0.23
T1 vs T2 × Group × Condition									0.12	0.32
T1 vs T3 × Group										
T1 vs T3 × Condition										
T1 vs T3 × Group × Condition										
Variance DV L1	0.30***	0.02	0.25***	0.02	0.25***	0.03	0.24***	0.03	0.24***	0.03
Variance DV L2	1.11***	0.26	0.81***	0.17	0.84***	0.18	0.83***	0.21	0.83***	0.21
Variance DV L3	0.02	0.16	0.12	0.13	0.11	0.14	0.12	0.14	0.12	0.15
Variance random L2					0.004	0.13	0.003	0.14	0.003	0.15
Variance random L3					0.001	0.09	0.001	0.09	0.001	0.10
Variance group							0.004	0.38	0.004	0.10
AIC	888.182		828.764		834.698		839.341		843.060	
BIC	903.693		859.786		877.353		897.507		908.982	

Supplementary Table 18. ***Continued.***

	Model 6		Model 7		Model 8	
	B	SE	B	SE	B	SE
Intercept	3.47***	0.16	3.37***	0.19	3.34***	0.20
T1 vs T2	-0.36***	0.08	-0.36***	0.09	-0.36***	0.09
T1 vs T3	-0.40***	0.07	-0.33**	0.12	-0.26†	0.15
Group (0 = R 1 = L)	-0.93***	0.21	-0.80*	0.33	-0.72*	0.32
Condition (0 = C 1 = E)	-0.16	0.22	0.02	0.25	0.09	0.27
Group × Condition			-0.23	0.43	-0.37	0.46
T1 vs T2 × Group						
T1 vs T2 × Condition						
T1 vs T2 × Group × Condition						
T1 vs T3 × Group			0.004	0.13	-0.15	0.24
T1 vs T3 × Condition			-0.13	0.14	-0.27	0.18
T1 vs T3 × Group × Condition					0.29	0.30
Variance DV L1	0.24***	0.03	0.24***	0.04	0.24***	0.04
Variance DV L2	0.86***	0.20	0.85***	0.22	0.85***	0.22
Variance DV L3	0.11	0.14	0.12	0.14	0.12	0.14
Variance random L2	0.01	0.11	0.01	0.11	0.010	0.12
Variance random L3	0.004	0.06	0.003	0.06	0.002	0.07
Variance group			0.01	0.34	0.004	0.34
AIC	833.684		839.981		842.371	
BIC	876.339		898.147		908.293	

Note. $ICC_{Level\ 3}$ = 0.024, $ICC_{Level\ 2}$ = 0.770. ****p* <.001; ***p* < .01, *, *p* <.05, †*p* <.10.

Supplementary Table 19. Full results for outgroup stereotype: Intolerant.

	Model 1		Model 2		Model 3		Model 4		Model 5	
	B	SE	B	SE	B	SE	B	SE	B	SE
Intercept	4.19***	0.15	5.08***	0.26	5.08***	0.26	4.97***	0.26	4.97***	0.25
T1 vs T2			-0.82***	0.16	-0.82***	0.18	-0.86**	0.32	-0.84*	0.35
T1 vs T3			-0.45	0.16	-0.45**	0.15	-0.45**	0.16	-0.45**	0.17
Group (0 = R 1 = L)			-0.40†	0.24	-0.43†	0.24	-0.28	0.42	-0.27	0.42
Condition (0 = C 1 = E)			-0.51†	0.27	-0.48†	0.27	-0.19	0.32	-0.18	0.34
Group × Condition							-0.47	0.54	-0.49	0.55
T1 vs T2 × Group							0.35	0.31	0.31	0.42
T1 vs T2 × Condition							-0.26	0.36	-0.30	0.49
T1 vs T2 × Group × Condition									0.09	0.64
T1 vs T3 × Group										
T1 vs T3 × Condition										
T1 vs T3 × Group × Condition										
Variance DV L1	1.69***	0.13	1.52***	0.12	1.49***	0.18	1.47***	0.18	1.46***	0.18
Variance DV L2	1.17***	0.31	1.14***	0.30	1.03***	0.33	0.66†	0.36	0.67†	0.38
Variance DV L3	0.04	0.26	0.03	0.24	0.02	0.25	0.01	0.24	0.01	0.25
Variance random L2					0.10	0.52	0.10	0.53	0.09	0.72
Variance random L3					0.02	0.36	0.02	0.35	0.02	0.35
Variance group							0.73	0.55	0.72	0.56
AIC	1345.397		1320.907		1325.747		1327.113		1331.078	
BIC	1360.908		1351.929		1368.402		1385.279		1397.000	

Supplementary Table 19. ***Continued.***

	Model 6		Model 7		Model 8	
	B	SE	B	SE	B	SE
Intercept	5.08***	0.26	4.97***	0.27	4.89***	0.28
T1 vs T2	-0.82***	0.16	-0.82***	0.17	-0.82***	0.18
T1 vs T3	-0.45*	0.18	-0.47	0.31	-0.22	0.33
Group (0 = R 1 = L)	-0.40†	0.24	-0.12	0.45	0.06	0.48
Condition (0 = C 1 = E)	-0.50†	0.27	-0.36	0.32	-0.19	0.35
Group × Condition			-0.43	0.54	-0.78	0.61
T1 vs T2 × Group			-			
T1 vs T2 × Condition						
T1 vs T2 × Group × Condition						
T1 vs T3 × Group			-0.17	0.32	-0.68	0.44
T1 vs T3 × Condition			0.20	0.36	-0.28	0.48
T1 vs T3 × Group × Condition					0.97	0.65
Variance DV L1	1.51***	0.18	1.50***	0.18	1.48***	0.21
Variance DV L2	1.20**	0.42	0.80	0.50	0.80	0.50
Variance DV L3	0.02	0.25	0.01	0.24	0.01	0.25
Variance random L2	0.05	0.54	0.05	0.57	0.04	0.69
Variance random L3	0.02	0.25	0.01	0.29	0.01	0.29
Variance group			0.76	0.63	0.75	0.65
AIC	1327.053		1330.124		1331.022	
BIC	1369.708		1388.290		1396.943	

Note. $ICC_{Level\ 3}$ = 0.015, $ICC_{Level\ 2}$ = 0.402. ****p* <.001; ***p* < .01, *, *p* <.05, †*p* <.10.

Supplementary Table 20. Full results for outgroup stereotype: Intelligent.

	Model 1		Model 2		Model 3		Model 4		Model 5	
	B	SE	B	SE	B	SE	B	SE	B	SE
Intercept	4.71***	0.12	3.87***	0.18	3.89***	0.18	3.89***	0.19	3.94***	0.20
T1 vs T2			0.28*	0.14	0.28†	0.16	0.13	0.24	-0.15	0.27
T1 vs T3			0.45**	0.15	0.45**	0.14	0.45**	0.14	0.45**	0.14
Group (0 = R 1 = L)			0.89***	0.18	0.91***	0.19	1.03***	0.28	0.89**	0.30
Condition (0 = C 1 = E)			0.30	0.21	0.24	0.23	0.21	0.27	0.11	0.28
Group × Condition							-0.12	0.38	0.09	0.42
T1 vs T2 × Group							-0.16	0.24	0.38	0.35
T1 vs T2 × Condition							0.44	0.31	0.96*	0.41
T1 vs T2 × Group × Condition									-1.03†	0.55
T1 vs T3 × Group										
T1 vs T3 × Condition										
T1 vs T3 × Group × Condition										
Variance DV L1	0.99***	0.06	0.95***	0.07	0.87***	0.10	0.87***	0.10	0.84***	0.09
Variance DV L2	0.82***	0.21	0.60**	0.17	0.51*	0.24	0.51†	0.27	0.54*	0.26
Variance DV L3	0.01	0.13	0.02	0.12	0.02	0.16	0.02	0.14	0.01	0.14
Variance random L2					0.21	0.28	0.17	0.30	0.07	0.44
Variance random L3					0.01	0.23	0.01	0.25	0.01	0.28
Variance group							0.02	0.32	0.02	0.31
AIC	1165.276		1135.390		1139.183		1140.698		1138.267	
BIC	1180.776		1166.389		1185.682		1198.822		1204.141	

Supplementary Table 20. ***Continued.***

	Model 6		Model 7		Model 8	
	B	SE	B	SE	B	SE
Intercept	3.88***	0.19	3.81***	0.21	3.71***	0.21
T1 vs T2	0.28*	0.14	0.28*	0.14	0.28*	0.14
T1 vs T3	0.45**	0.16	0.51*	0.21	0.74***	0.24
Group (0 = R 1 = L)	0.87***	0.18	1.03***	0.31	1.22***	0.34
Condition (0 = C 1 = E)	0.31	0.21	0.37	0.30	0.56	0.35
Group × Condition			-0.17	0.38	-0.55	0.44
T1 vs T2 × Group						
T1 vs T2 × Condition						
T1 vs T2 × Group × Condition						
T1 vs T3 × Group			-0.19	0.31	-0.66	0.44
T1 vs T3 × Condition			0.06	0.30	-0.41	0.55
T1 vs T3 × Group × Condition					0.93	0.75
Variance DV L1	0.92***	0.09	0.92***	0.10	0.90***	0.10
Variance DV L2	0.71**	0.21	0.68*	0.27	0.69*	0.27
Variance DV L3	0.02	0.13	0.01	0.13	0.01	0.15
Variance random L2	0.06	0.34	0.05	0.34	0.04	0.46
Variance random L3	0.03	0.20	0.03	0.21	0.03	0.22
Variance group			0.03	0.33	0.02	0.34
AIC	1139.999		1146.866		1145.959	
BIC	1182.623		1204.990		1211.832	

Note. $ICC_{Level\ 3}$ = 0.008, $ICC_{Level\ 2}$ = 0.447. ***p <.001; **p < .01, *, p <.05, †p <.10.

Supplementary Table 21. Full results for meta-stereotype: Intolerant.

	Model 1		Model 2		Model 3		Model 4		Model 5	
	B	SE	B	SE	B	SE	B	SE	B	SE
Intercept	4.42***	0.15	4.40***	0.24	4.40***	0.27	4.23***	0.32	4.28***	0.32
T1 vs T2			-0.44**	0.17	-0.44*	0.22	-0.23	0.40	-0.35	0.43
T1 vs T3			-0.33†	0.18	-0.33†	0.17	-0.33†	0.18	-0.33†	0.20
Group (0 = R 1 = L)			0.50*	0.24	0.50*	0.25	0.65	0.41	0.55	0.43
Condition (0 = C 1 = E)			0.05	0.28	0.04	0.28	0.39	0.41	0.30	0.41
Group × Condition							-0.36	0.50	-0.18	0.56
T1 vs T2 × Group							0.11	0.35	0.35	0.51
T1 vs T2 × Condition							-0.50	0.41	-0.26	0.54
T1 vs T2 × Group × Condition									-0.46	0.73
T1 vs T3 × Group										
T1 vs T3 × Condition										
T1 vs T3 × Group × Condition										
Variance DV L1	2.08***	0.17	2.02***	0.18	1.87***	0.22	1.87***	0.23	1.86***	0.23
Variance DV L2	1.00**	0.35	0.96**	0,34	1.12*	0.45	1.00*	0.46	1.02*	0.47
Variance DV L3	0.03	0.22	0.04	0.21	0.04	0.26	0.03	0.25	0.03	0.25
Variance random L2					0.19	0.80	0.20	0.72	0.18	0.82
Variance random L3					0.25	0.52	0.18	0.46	0.17	0.48
Variance group							0.25	0.66	0.23	0.69
AIC	1390.850		1388.552		1395.453		1398.360		1401.785	
BIC	1406.360		1419.574		1441.986		1456.526		1467.707	

Supplementary Table 21. ***Continued*.**

	Model 6		Model 7		Model 8	
	B	SE	B	SE	B	SE
Intercept	4.37***	0.25	4.17***	0.31	4.09***	0.33
T1 vs T2	-0.44*	0.18	-0.44*	0.19	-0.44*	0.18
T1 vs T3	-0.33	0.21	0.04	0.39	0.33	0.45
Group (0 = R 1 = L)	0.51*	0.24	0.76†	0.39	0.92*	0.41
Condition (0 = C 1 = E)	0.08	0.29	0.42	0.38	0.58	0.45
Group × Condition			-0.40	0.51	-0.71	0.55
T1 vs T2 × Group						
T1 vs T2 × Condition						
T1 vs T2 × Group × Condition						
T1 vs T3 × Group			-0.17	0.38	-0.76	0.57
T1 vs T3 × Condition			-0.53	0.39	-1.10†	0.66
T1 vs T3 × Group × Condition					1.13	0.90
Variance DV L1	1.95***	0.27	1.94***	0.28	1.89***	0.27
Variance DV L2	0.89*	0.45	0.79	0.49	0.83	0.50
Variance DV L3	0.03	0.25	0.02	0.23	0.02	0.23
Variance random L2	0.11	0.93	0.11	0.84	0.07	1.23
Variance random L3	0.12	0.52	0.07	0.43	0.06	0.55
Variance group			0.18	0.63	0.17	0.64
AIC	1395.631		1397.847		1398.351	
BIC	1442.164		1456.013		1464.273	

Note. $ICC_{Level\ 3}$ = 0.012, $ICC_{Level\ 2}$ = 0.321. ****p* <.001; ***p* < .01, *, *p* <.05, †*p* <.10.

Supplementary Table 22. Full results for meta-stereotype: Intelligent.

	Model 1		Model 2		Model 3		Model 4		Model 5	
	B	SE	B	SE	B	SE	B	SE	B	SE
Intercept	3.67***	0.12	3.73***	0.23	3.74***	0.24	3.76***	0.27	3.76***	0.28
T1 vs T2			0.37*	0.16	0.37*	0.17	0.01	0.32	-0.003	0.39
T1 vs T3			0.64***	0.15	0.64***	0.14	0.64***	0.15	0.64***	0.15
Group (0 = R 1 = L)			-0.71**	0.23	-0.71**	0.22	-0.57†	0.33	-0.58†	0.35
Condition (0 = C 1 = E)			-0.07	0.22	-0.09	0.23	-0.10	0.31	-0.11	0.33
Group × Condition							-0.33	0.46	-0.31	0.50
T1 vs T2 × Group							0.11	0.28	0.13	0.40
T1 vs T2 × Condition							0.59†	0.32	0.61	0.46
T1 vs T2 × Group × Condition									-0.05	0.61
T1 vs T3 × Group										
T1 vs T3 × Condition										
T1 vs T3 × Group × Condition										
Variance DV L1	1.28***	0.09	1.18***	0.08	1.08***	0.13	1.08***	0.14	1.07***	0.14
Variance DV L2	1.05***	0.28	0.99***	0.28	0.99**	0.32	0.68†	0.38	0.70†	0.38
Variance DV L3	0.03	0.24	0.001	0.21	0.05	0.22	0.05	0.21	0.05	0.22
Variance random L2					0.25	0.47	0.19	0.47	0.14	0.59
Variance random L3					0.05	0.30	0.03	0.28	0.03	0.29
Variance group							0.58	0.51	0.56	0.52
AIC	1256.620		1234.118		1239.541		1238.852		1242.576	
BIC	1272.120		1265.117		1282.165		1296.976		1308.450	

Supplementary Table 22. ***Continued*****.**

	Model 6		Model 7		Model 8	
	B	SE	B	SE	B	SE
Intercept	3.71***	0.24	3.55***	0.27	3.60***	0.28
T1 vs T2	0.37*	0.15	0.37*	0.16	0.37*	0.18
T1 vs T3	0.64***	0.16	0.94**	0.27	0.78*	0.35
Group (0 = R 1 = L)	-0.70**	0.22	-0.52	0.36	-0.61	0.43
Condition (0 = C 1 = E)	-0.04	0.22	0.23	0.32	0.14	0.35
Group × Condition			-0.29	0.48	-0.12	0.55
T1 vs T2 × Group						
T1 vs T2 × Condition						
T1 vs T2 × Group × Condition						
T1 vs T3 × Group			-0.10	0.33	0.22	0.50
T1 vs T3 × Condition			-0.47†	0.28	-0.16	0.62
T1 vs T3 × Group × Condition					-0.62	0.91
Variance DV L1	1.14***	0.14	1.13***	0.14	1.12***	0.14
Variance DV L2	0.88*	0.35	0.60†	0.36	0.60	0.41
Variance DV L3	0.06	0.25	0.05	0.24	0.05	0.24
Variance random L2	0.07	0.62	0.06	0.62	0.05	0.68
Variance random L3	0.06	0.29	0.04	0.28	0.03	0.29
Variance group			0.56	0.54	0.56	0.64
AIC	1238.556		1240.764		1243.156	
BIC	1282.180		1298.888		1309.030	

Note. $ICC_{Level\ 3}$ = 0.016, $ICC_{Level\ 2}$ = 0.442. ***p <.001; **p < .01, *, p <.05, †p <.10.

Supplementary Table 23. Full results for meta-emotions.

	Model 1		Model 2		Model 3		Model 4		Model 5	
	B	SE	B	SE	B	SE	B	SE	B	SE
Intercept	4.34***	0.12	4.63***	0.21	4.62***	0.23	4.62***	0.20	4.57***	0.20
T1 vs T2			-0.59***	0.13	-0.59***	0.18	-0.51	0.31	-0.36	0.34
T1 vs T3			-0.69***	0.18	-0.69***	0.12	-0.69***	0.12	-0.69***	0.13
Group (0 = R 1 = L)			0.44*	0.22	0.44†	0.24	0.38	0.30	0.47	0.30
Condition (0 = C 1 = E)			-0.18	0.24	-0.15	0.25	-0.12	0.30	-0.03	0.31
Group × Condition							0.04	0.44	-0.13	0.44
T1 vs T2 × Group							0.12	0.28	-0.17	0.43
T1 vs T2 × Condition							-0.27	0.29	-0.55	0.41
T1 vs T2 × Group × Condition									0.57	0.58
T1 vs T3 × Group										
T1 vs T3 × Condition										
T1 vs T3 × Group × Condition										
Variance DV L1	1.06***	0.08	0.92***	0.07	0.60***	0.07	0.60***	0.07	0.60***	0.07
Variance DV L2	0.93**	0.27	0.89**	0.27	1.05***	0.29	0.76*	0.31	0.77*	0.32
Variance DV L3	0.18	0.23	0.22	0.26	0.18	0.26	0.19	0.25	0.19	0.25
Variance random L2					0.76*	0.38	0.72*	0.28	0.57	0.36
Variance random L3					0.20	0.35	0.19	0.25	0.24	0.27
Variance group							0.22	0.27	0.57	0.41
AIC	1210.564		1180.269		1171.461		1173.640		1175.843	
BIC	1226.075		1211.291		1217.994		1231.806		1241.764	

Supplementary Table 23. *Continued*.

	Model 6		Model 7		Model 8	
	B	SE	B	SE	B	SE
Intercept	4.63***	0.23	4.65***	0.21	4.68***	0.21
T1 vs T2	-0.59***	0.13	-0.58***	0.14	-0.58***	0.14
T1 vs T3	-0.69***	0.21	-0.68*	0.30	-0.77*	0.31
Group (0 = R 1 = L)	0.45*	0.21	0.42	0.31	0.37	0.34
Condition (0 = C 1 = E)	-0.18	0.25	-0.24	0.30	-0.30	0.32
Group × Condition			0.07	0.42	0.18	0.50
T1 vs T2 × Group						
T1 vs T2 × Condition						
T1 vs T2 × Group × Condition						
T1 vs T3 × Group			-0.07	0.26	0.11	0.43
T1 vs T3 × Condition			0.07	0.31	0.23	0.40
T1 vs T3 × Group × Condition					-0.34	0.60
Variance DV L1	0.91***	0.09	0.92***	0.09	0.91***	0.09
Variance DV L2	0.82*	0.41	0.56	0.38	0.57	0.39
Variance DV L3	0.29	0.32	0.23	0.28	0.23	0.28
Variance random L2	0.02	0.31	0.02	0.29	0.01	0.47
Variance random L3	0.02	0.25	0.01	0.23	0.004	0.24
Variance group			0.61	0.42	0.61	0.43
AIC	1187.986		1191.576		1194.999	
BIC	1234.519		1249.742		1260.921	

Note. $ICC_{Level\ 3}$ = 0.096, $ICC_{Level\ 2}$ = 0.415. ****p* <.001; ***p* < .01, *, *p* <.05, †*p* <.10.

Supplementary Table 24. Full results for rationality attributions.

	Model 1		Model 2		Model 3		Model 4		Model 5	
	B	SE	B	SE	B	SE	B	SE	B	SE
Intercept	4.27***	0.11	3.46***	0.20	3.46***	0.22	3.41***	0.20	3.49***	0.21
T1 vs T2			0.65***	0.11	0.65***	0.12	0.60**	0.20	0.38†	0.21
T1 vs T3			0.37***	0.10	0.37***	0.10	0.37***	0.11	0.37***	0.10
Group (0 = R 1 = L)			0.81***	0.20	0.81***	0.21	1.01**	0.31	0.85**	0.29
Condition (0 = C 1 = E)			0.14	0.25	0.15	0.30	0.16	0.36	0.01	0.38
Group × Condition							-0.24	0.46	0.07	0.46
T1 vs T2 × Group							-0.22	0.19	0.21	0.27
T1 vs T2 × Condition							0.30	0.22	0.72*	0.30
T1 vs T2 × Group × Condition									-0.83*	0.39
T1 vs T3 × Group										
T1 vs T3 × Condition										
T1 vs T3 × Group × Condition										
Variance DV L1	0.72***	0.06	0.61***	0.06	0.58***	0.08	0.57***	0.09	0.56***	0.08
Variance DV L2	1.02***	0.26	0.87***	0.24	0.91**	0.30	0.82**	0.31	0.83*	0.33
Variance DV L3	0.02	0.17	0.05	0.17	0.04	0.19	0.03	0.20	0.03	0.22
Variance random L2					0.11	0.25	0.08	0.25	0.05	0.29
Variance random L3					0.04	0.19	0.01	0.16	0.01	0.17
Variance group L3							0.19	0.35	0.18	0.36
AIC	1104.144		1057.241		1064.939		1065.810		1064.128	
BIC	1119.655		1088.263		1111.472		1123.976		1130.050	

Supplementary Table 24. *Continued.*

	Model 6		Model 7		Model 8	
	B	SE	B	SE	B	SE
Intercept	3.45***	0.20	3.47***	0.21	3.43***	0.22
T1 vs T2	0.65***	0.12	0.65***	0.12	0.65***	0.13
T1 vs T3	0.37***	0.10	0.18	0.19	0.28	0.22
Group (0 = R 1 = L)	0.82***	0.22	0.86**	0.32	0.94**	0.35
Condition (0 = C 1 = E)	0.15	0.26	0.16	0.37	0.24	0.38
Group × Condition			-0.19	0.47	-0.34	0.53
T1 vs T2 × Group						
T1 vs T2 × Condition						
T1 vs T2 × Group × Condition						
T1 vs T3 × Group					-0.06	0.31
T1 vs T3 × Condition					0.02	0.35
T1 vs T3 × Group × Condition					0.40	0.47
Variance DV L1	0.60***	0.08	0.60***	0.08	0.60***	0.09
Variance DV L2	0.92**	0.28	0.82**	0.29	0.83**	0.31
Variance DV L3	0.03	0.22	0.03	0.20	0.03	0.21
Variance random L2	0.02	0.24	0.02	0.22	0.02	0.27
Variance random L3	0.01	0.19	0.01	0.18	0.01	0.19
Variance group			0.18	0.34	0.18	0.35
AIC	1065.204		1068.411		1071.170	
BIC	1111.736		1126.577		1137.091	

Note: $ICC_{Level\ 3}$ = 0.011 $ICC_{Level\ 2}$ = 0.580. ****p* <.001; ***p* < .01, *, *p* <.05, †*p* <.10.

Supplementary Table 25. Full results for emotionality attributions.

	Model 1		Model 2		Model 3		Model 4		Model 5	
	B	SE	B	SE	B	SE	B	SE	B	SE
Intercept	4.82***	0.14	5.38***	0.25	5.33***	0.25	5.31***	0.25	5.28***	0.26
T1 vs T2			-0.54***	0.12	-0.54**	0.17	-0.19	0.33	-0.08	0.40
T1 vs T3			-0.04	0.18	-0.04	0.15	-0.04	0.15	-0.04	0.15
Group (0 = R 1 = L)			-0.79***	0.21	-0.79***	0.20	-0.88**	0.33	-0.82*	0.34
Condition (0 = C 1 = E)			0.06	0.26	0.16	0.28	0.19	0.33	0.25	0.35
Group × Condition							0.18	0.43	0.06	0.44
T1 vs T2 × Group							-0.04	0.27	-0.24	0.45
T1 vs T2 × Condition							-0.65*	0.31	-0.85†	0.47
T1 vs T2 × Group × Condition									0.40	0.60
T1 vs T3 × Group										
T1 vs T3 × Condition										
T1 vs T3 × Group × Condition										
Variance DV L1	1.10***	0.06	1.01***	0.06	0.88***	0.08	0.87***	0.08	0.83***	0.07
Variance DV L2	1.04***	0.20	0.91***	0.18	0.88***	0.21	0.65**	0.24	0.69***	0.26
Variance DV L3	0.01	0.22	0.02	0.20	0.01	0.26	0.01	0.21	0.01	0.22
Variance random L2					0.07	0.34	0.06	0.29	0.03	0.32
Variance random L3					0.34	0.34	0.28	0.29	0.18	0.31
Variance group							0.49	0.36	0.45	0.36
AIC	1217.339		1190.317		1191.545		1188.711		1189.849	
BIC	1232.850		1221.339		1238.078		1246.877		1255.771	

Supplementary Table 25. ***Continued.***

	Model 6		Model 7		Model 8	
	B	SE	B	SE	B	SE
Intercept	5.38***	0.26	5.46***	0.28	5.47***	0.29
T1 vs T2	-0.54***	0.14	-0.54***	0.14	-0.54***	0.15
T1 vs T3	-0.04	0.19	-0.14	0.40	-0.16	0.47
Group (0 = R 1 = L)	-0.79***	0.21	-0.87**	0.32	-0.88**	0.32
Condition (0 = C 1 = E)	0.06	0.31	-0.13	0.36	-0.14	0.39
Group × Condition			0.19	0.42	0.21	0.47
T1 vs T2 × Group						
T1 vs T2 × Condition						
T1 vs T2 × Group × Condition						
T1 vs T3 × Group			-0.09	0.35	-0.05	0.48
T1 vs T3 × Condition			0.29	0.34	0.32	0.58
T1 vs T3 × Group × Condition					-0.07	0.70
Variance DV L1	1.01***	0.09	1.00***	0.09	1.00***	0.09
Variance DV L2	0.92***	0.31	0.67*	0.31	0.68*	0.33
Variance DV L3	0.01	0.27	0.01	0.20	0.01	0.20
Variance random L2	0.01	0.57	0.01	0.58	0.01	0.77
Variance random L3	0.01	0.27	0.01	0.38	0.004	0.45
Variance group			0.49	0.37	0.49	0.37
AIC	1198.589		1200.417		1204.446	
BIC	1245.122		1258.583		1270.367	

Note. $ICC_{Level\ 3}$ = .075 $ICC_{Level\ 2}$ = .410. ***p <.001; **p < .01, *, p <.05, †p <.10.

Supplementary Table 26. Full results for externality attributions.

	Model 1		Model 2		Model 3		Model 4		Model 5	
	B	SE	B	SE	B	SE	B	SE	B	SE
Intercept	4.95***	0.11	5.47***	0.20	5.40***	0.20	5.28***	0.20	5.25***	0.20
T1 vs T2			-0.50***	0.11	-0.51***	0.14	-0.19	0.27	0.04	0.32
T1 vs T3			-0.03	0.15	-0.03	0.11	-0.03	0.11	-0.03	0.11
Group (0 = R 1 = L)			-0.52**	0.18	-0.52**	0.17	-0.34	0.27	-0.28	0.27
Condition (0 = C 1 = E)			-0.16	0.22	-0.03	0.24	0.21	0.32	0.26	0.33
Group × Condition							-0.34	0.39	-0.46	0.39
T1 vs T2 × Group							-0.01	0.22	-0.48	0.38
T1 vs T2 × Condition							-0.59*	0.28	-1.03**	0.41
T1 vs T2 × Group × Condition									0.89†	0.49
T1 vs T3 × Group										
T1 vs T3 × Condition										
T1 vs T3 × Group × Condition										
Variance DV L1	0.87***	0.07	0.79***	0.06	0.57***	0.07	0.57***	0.07	0.57***	0.07
Variance DV L2	0.71**	0.23	0.60**	0.20	0.57**	0.18	0.52*	0.25	0.51†	0.26
Variance DV L3	0.10	0.16	0.16	0.16	0.14	0.19	0.15	0.19	0.15	0.19
Variance random L2							0.55†	0.31	0.31	0.39
Variance random L3							0.02	0.29	0.02	0.29
Variance group							0.07	0.27	0.07	0.27
AIC	1128.575		1104.201		1096.442		1094.591		1093.235	
BIC	1144.086		1135.223		1142.975		1152.757		1159.156	

Supplementary Table 26. ***Continued*****.**

	Model 6		Model 7		Model 8	
	B	SE	B	SE	B	SE
Intercept	5.47***	0.21	5.42***	0.23	5.44***	0.24
T1 vs T2	-0.50***	0.11	-0.50***	0.11	-0.50***	0.11
T1 vs T3	-0.03	0.16	-0.003	0.27	-0.07	0.31
Group (0 = R 1 = L)	-0.52**	0.18	-0.40	0.29	-0.44	0.32
Condition (0 = C 1 = E)	-0.16	0.22	-0.10	0.30	-0.14	0.32
Group × Condition			-0.17	0.36	-0.08	0.46
T1 vs T2 × Group						
T1 vs T2 × Condition						
T1 vs T2 × Group × Condition						
T1 vs T3 × Group			-0.10	0.28	0.03	0.39
T1 vs T3 × Condition			0.05	0.30	0.18	0.41
T1 vs T3 × Group × Condition					-0.25	0.66
Variance DV L1	0.79***	0.07	0.78***	0.07	0.78***	0.07
Variance DV L2	0.60*	0.21	0.56†	0.28	0.57†	0.31
Variance DV L3	0.17	0.22	0.16	0.18	0.16	0.20
Variance random L2	0.01	0.31	0.01	0.33	0.01	0.49
Variance random L3	0.17	0.22	0.003	0.29	0.003	0.33
Variance group			0.09	0.29	0.08	0.30
AIC	1112.611		1118.051		1121.673	
BIC	1159.144		1176.217		1187.595	

Note. $ICC_{Level\ 3}$ = .075 $ICC_{Level\ 2}$ = .410. ****p* <.001; ***p* < .01, *, *p* <.05, †*p* <.10.

Supplementary Table 27: Full results for contempt.

	Model 1		Model 2		Model 3		Model 4		Model 5	
	B	SE	B	SE	B	SE	B	SE	B	SE
Intercept	2.61***	0.14	3.28***	0.16	3.28***	0.16	3.24***	0.18	3.22***	0.19
T1 vs T2			-0.44***	0.09	-0.44***	0.10	-0.40*	0.18	-0.29	0.20
T1 vs T3			-0.15†	0.09	-0.15	0.09	-0.15	0.10	-0.15	0.10
Group (0 = R 1 = L)					-0.86***	0.22	-0.88*	0.35	-0.83*	0.33
Condition (0 = C 1 = E)					-0.07	0.23	0.06	0.26	0.10	0.29
Group × Condition							-0.09	0.50	-0.18	0.48
T1 vs T2 × Group							0.26	0.19	0.06	0.25
T1 vs T2 × Condition							-0.34†	0.19	-0.53*	0.26
T1 vs T2 × Group × Condition									0.38	0.39
0T1 vs T3 × Group										
T1 vs T3 × Condition										
T1 vs T3 × Group × Condition										
Variance DV L1	0.50***	0.04	0.45***	0.04	0.44***	0.05	0.43***	0.05	0.43***	0.05
Variance DV L2	1.04***	0.25	0.78***	0.21	0.74**	0.24	0.74**	0.24	0.74**	0.23
Variance DV L3	0.04	0.15	0.13	0.15	0.13	0.18	0.13	0.17	0.13	0.17
Variance random T L2					0.03	0.19	0.02	0.18	0.02	0.18
Variance random T L3					0.01	0.12	0.004	0.12	0.003	0.12
Variance random Group							0.01	0.40	0.01	0.42
AIC	1014.175		977.085		982.490		982.490		984.855	
BIC	1029.686		1008.106		1040.656		1040.656		1050.776	

Supplementary Table 27. ***Continued*.**

	Model 6		Model 7		Model 8	
	B	SE	B	SE	B	SE
Intercept	3.28***	0.17	3.27***	0.19	3.27***	0.20
T1 vs T2	-0.44***	0.09	-0.44***	0.09	-0.44***	0.09
T1 vs T3	-0.15	0.09	-0.15	0.18	-0.14	0.23
Group (0 = R 1 = L)	-0.83***	0.24	-0.77*	0.36	-0.77*	0.38
Condition (0 = C 1 = E)	-0.10	0.24	-0.10	0.26	-0.10	0.27
Group × Condition			-0.06	0.46	-0.06	0.53
T1 vs T2 × Group						
T1 vs T2 × Condition						
T1 vs T2 × Group × Condition						
T1 vs T3 × Group			-0.10	0.22	-0.11	0.38
T1 vs T3 × Condition			0.09	0.20	0.09	0.35
T1 vs T3 × Group × Condition					0.02	0.53
Variance DV L1	0.45***	0.06	0.45***	0.06	0.45***	0.06
Variance DV L2	0.74**	0.22	0.75**	0.25	0.75**	0.26
Variance DV L3	0.15	0.16	0.13	0.17	0.13	0.17
Variance random L2	0.01	0.23	0.01	0.22	0.01	0.29
Variance random L3	0.01	0.12	0.004	0.13	0.003	0.14
Variance group			0.01	0.37	0.01	0.38
AIC	985.069		990.545		994.598	
BIC	1031.602		1048.711		1060.519	

Note. $ICC_{Level\ 3}$ = .024, $ICC_{Level\ 2}$ = .657. ****p* <.001; ***p* < .01, *, *p* <.05, †*p* <.10.

Supplementary Table 28. Full results for willingness to compromise.

	Model 1		Model 2		Model 3		Model 4		Model 5	
	B	SE	B	SE	B	SE	B	SE	B	SE
Intercept	5.38***	0.11	5.31***	0.20	5.31***	0.21	5.37***	0.18	5.37***	0.18
T1 vs T2			0.39***	0.08	0.39***	0.10	0.20	0.16	0.20	0.17
T1 vs T3			0.21*	0.10	0.21*	0.10	0.21*	0.10	0.21*	0.10
Group (0 = R 1 = L)			-0.33†	0.19	-0.33†	0.19	-0.33	0.32	-0.33	0.34
Condition (0 = C 1 = E)			0.06	0.21	0.07	0.25	-0.05	0.25	-0.05	0.26
Group × Condition							-0.01	0.42	-0.01	0.47
T1 vs T2 × Group							0.02	0.19	0.02	0.27
T1 vs T2 × Condition							0.35*	0.18	0.35	0.28
T1 vs T2 × Group × Condition									0.01	0.42
T1 vs T3 × Group										
T1 vs T3 × Condition										
T1 vs T3 × Group × Condition										
Variance DV L1	0.42***	0.03	0.38***	0.03	0.38***	0.04	0.37***	0.05	0.37***	0.05
Variance DV L2	0.63***	0.15	0.59**	0.18	0.59**	0.19	0.31†	0.17	0.31†	0.18
Variance DV L3	0.10	0.13	0.12	0.13	0.13	0.14	0.13	0.14	0.13	0.14
Variance random L2					0.01	0.17	0.01	0.17	0.01	0.21
Variance random L3					0.02	0.11	0.01	0.12	0.01	0.12
Variance group							0.56†	0.30	0.56†	0.33
AIC	929.571		910.298		918.542		911.428		915.485	
BIC	945.082		941.320		965.075		969.594		981.406	

Supplementary Table 28. ***Continued*.**

	Model 6		Model 7		Model 8	
	B	SE	B	SE	B	SE
Intercept	5.30***	0.20	5.29***	0.17	5.32***	0.19
T1 vs T2	0.39***	0.08	0.39***	0.08	0.39***	0.08
T1 vs T3	0.21†	0.12	0.30	0.22	0.19	0.29
Group (0 = R 1 = L)	-0.30	0.21	-0.28	0.32	-0.33	0.35
Condition (0 = C 1 = E)	0.06	0.21	0.03	0.23	-0.01	0.25
Group × Condition			-0.04	0.40	0.13	0.44
T1 vs T2 × Group						
T1 vs T2 × Condition						
T1 vs T2 × Group × Condition						
T1 vs T3 × Group			-0.20	0.18	0.02	0.31
T1 vs T3 × Condition			0.03	0.21	0.24	0.35
T1 vs T3 × Group × Condition					-0.43	0.38
Variance DV L1	0.37***	0.04	0.37***	0.04	0.37***	0.04
Variance DV L2	0.53**	0.17	0.33†	0.17	0.33†	0.18
Variance DV L3	0.11	0.14	0.10	0.16	0.10	0.16
Variance random L2	0.02	0.19	0.02	0.18	0.02	0.22
Variance random L3	0.01	0.15	0.002	0.14	0.002	0.14
Variance group			0.47	0.32	0.48	0.34
AIC	914.715		914.407		916.076	
BIC	961.248		972.573		981.997	

Note. DV: Coop $ICC_{Level\ 3}$ = .104 $ICC_{Level\ 2}$ = .532. ****p* <.001; ***p* < .01, *, *p* <.05, †*p* <.10.

Supplementary Table 29. Full results for collective action intentions.

	Model 1		Model 2		Model 3		Model 4		Model 5	
	B	SE	B	SE	B	SE	B	SE	B	SE
Intercept	3.66***	0.16	4.05***	0.28	4.04***	0.28	3.69***	0.35	3.75***	0.36
T1 vs T2			-0.28*	0.13	-0.29*	0.14	-0.16	0.25	-0.37	0.30
T1 vs T3			-0.25	0.17	-0.25	0.17	-0.25	0.21	-0.25	0.22
Group (0 = R 1 = L)			-0.49	0.34	-0.48	0.33	0.20	0.51	0.08	0.51
Condition (0 = C 1 = E)			0.06	0.33	0.07	0.36	0.71	0.48	0.59	0.51
Group × Condition							-1.23†	0.71	-1.00	0.71
T1 vs T2 × Group							-0.15	0.28	0.26	0.49
T1 vs T2 × Condition							-0.09	0.31	0.30	0.43
T1 vs T2 × Group × Condition									-0.77	0.60
T1 vs T3 × Group										
T1 vs T3 × Condition										
T1 vs T3 × Group × Condition										
Variance DV L1	1.01***	0.05	0.98***	0.06	0.91***	0.10	0.91***	0.11	0.91***	0.11
Variance DV L2	2.48***	0.61	2.37***	0.60	2.29**	0.73	2.16*	0.87	2.17*	0.90
Variance DV L3	0.04	0.35	0.10	0.37	0.15	0.41	0.20	0.37	0.20	0.39
Variance random L2					0.19	0.36	0.18	0.36	0.12	0.40
Variance random L3					0.02	0.34	0.02	0.30	0.02	0.30
Variance group							0.04	1.06	0.04	1.08
AIC	1278.755		1278.353		1285.685		1287.001		1288.137	
BIC	1294.266		1309.375		1332.218		1345.167		1354.058	

Supplementary Table 29. ***Continued*****.**

	Model 6		Model 7		Model 8	
	B	SE	B	SE	B	SE
Intercept	4.05***	0.29	3.72***	0.34	3.71***	0.34
T1 vs T2	-0.28†	0.15	-0.28†	0.16	-0.28†	0.16
T1 vs T3	-0.25	0.20	-0.26	0.30	-0.21	0.33
Group (0 = R 1 = L)	-0.48	0.35	0.20	0.51	0.22	0.52
Condition (0 = C 1 = E)	0.05	0.34	0.67	0.48	0.69	0.48
Group × Condition			-1.28†	0.68	-1.33†	0.69
T1 vs T2 × Group						
T1 vs T2 × Condition						
T1 vs T2 × Group × Condition						
T1 vs T3 × Group			-0.08	0.25	-0.18	0.42
T1 vs T3 × Condition			0.10	0.38	-0.01	0.46
T1 vs T3 × Group × Condition					0.21	0.53
Variance DV L1	0.97***	0.11	0.97***	0.11	0.95***	0.11
Variance DV L2	2.26***	0.61	2.09**	0.75	2.11**	0.80
Variance DV L3	0.12	0.43	0.18	0.37	0.18	0.38
Variance random L2	0.04	0.42	0.04	0.45	0.03	0.53
Variance random L3	0.01	0.32	0.01	0.31	0.01	0.35
Variance group			0.03	1.08	0.03	1.13
AIC	1286.132		1287.261		1290.829	
BIC	1332.665		1345.427		1356.751	

Note. DV: Solid $ICC_{Level\ 3}$ = 0.049, $ICC_{Level\ 2}$ = 0.667. ****p* <.001; ***p* < .01, *, *p* <.05, †*p* <.10.

Supplementary Table 30. Full results for anti-Brexit opinions.

	Model 1		Model 2		Model 3		Model 4		Model 5	
	B	SE	B	SE	B	SE	B	SE	B	SE
Intercept	3.62***	0.21	4.89***	0.15	4.89***	0.15	5.00***	0.17	4.95***	0.17
T1 vs T2			-0.19**	0.06	-0.19**	0.07	-0.21	0.14	-0.10	0.15
T1 vs T3			-0.10†	0.06	-0.10	0.06	-0.10	0.06	-0.10	0.06
Group (0 = R 1 = L)			-2.24***	0.18	-2.23***	0.19	-2.50***	0.27	-2.41***	0.26
Condition (0 = C 1 = E)			-0.12	0.17	-0.11	0.18	-0.26	0.23	-0.18	0.24
Group × Condition							0.39	0.38	0.21	0.43
T1 vs T2 × Group							0.20	0.16	-0.04	0.19
T1 vs T2 × Condition							-0.14	0.14	-0.36†	0.19
T1 vs T2 × Group × Condition									0.46	0.28
0T1 vs T3 × Group										
T1 vs T3 × Condition										
T1 vs T3 × Group × Condition										
Variance DV L1	0.21***	0.01	0.20***	0.01	0.20***	0.02	0.20***	0.02	0.19***	0.02
Variance DV L2	1.99***	0.41	0.68***	0.15	0.67***	0.18	0.67**	0.22	0.67**	0.22
Variance DV L3	0.01	0.38	0.06	0.12	0.07	0.14	0.08	0.14	0.07	0.14
Variance random T L2					0.01	0.06	0.004	0.07	0.003	0.09
Variance random T L3					0.002	0.05	0.001	0.05	0.001	0.06
Variance random Group							0.01	0.33	0.01	0.33
AIC	875.531		760.089		768.324		766.897		765.594	
BIC	891.075		791.178		814.957		825.188		831.658	

Supplementary Table 30. ***Continued*****.**

	Model 6		Model 7		Model 8	
	B	SE	B	SE	B	SE
Intercept	4.91***	0.15	5.00***	0.16	5.00***	0.16
T1 vs T2	-0.19**	0.06	-0.19**	0.06	-0.19**	0.07
T1 vs T3	-0.10	0.06	-0.14	0.11	-0.14	0.14
Group (0 = R 1 = L)	-2.28***	0.18	-2.47***	0.23	-2.47***	0.24
Condition (0 = C 1 = E)	-0.10	0.20	-0.28	0.22	-0.28	0.23
Group × Condition			0.37	0.36	-0.37	0.38
T1 vs T2 × Group						
T1 vs T2 × Condition						
T1 vs T2 × Group × Condition						
T1 vs T3 × Group			0.12	0.15	0.13	0.23
T1 vs T3 × Condition			-0.05	0.14	-0.05	0.23
T1 vs T3 × Group × Condition					-0.01	0.31
Variance DV L1	0.20***	0.02	0.19**	0.02	0.19***	0.02
Variance DV L2	0.58**	0.18	0.57**	0.20	0.57**	0.21
Variance DV L3	0.09	0.14	0.10	0.13	0.10	0.13
Variance random L2	0.02	0.06	0.02	0.06	0.02	0.08
Variance random L3	0.002	0.04	0.001	0.04	0.001	0.05
Variance group			0.01	0.29	0.01	0.30
AIC	762.624		765.549		769.594	
BIC	809.257		823.841		835.657	

Note: $ICC_{Level\ 3}$ = 0.003 $ICC_{Level\ 2}$ = 0.900. ***p <.001; **p < .01, *, p <.05, †p <.10.

Supplementary Table 31. Full results for intergroup anxiety.

	Model 1		Model 2		Model 3		Model 4		Model 5	
	B	SE	B	SE	B	SE	B	SE	B	SE
Intercept	3.77***	0.13	4.06***	0.26	4.07***	0.27	3.97***	0.19	3.92***	0.20
T1 vs T2			-0.04	0.12	-0.04	0.15	-0.15	0.27	0.004	0.24
T1 vs T3			-0.12	0.13	-0.12	0.11	-0.12	0.12	-0.12	0.12
Group (0 = R 1 = L)			-0.44†	0.27	-0.46†	0.27	-0.26	0.40	-0.17	0.40
Condition (0 = C 1 = E)			-0.03	0.25	-0.03	0.26	0.21	0.27	0.30	0.29
Group × Condition							-0.49	0.52	-0.66	0.56
T1 vs T2 × Group							0.21	0.28	-0.10	0.36
T1 vs T2 × Condition							0.02	0.27	-0.28	0.30
T1 vs T2 × Group × Condition									0.60	0.49
T1 vs T3 × Group										
T1 vs T3 × Condition										
T1 vs T3 × Group × Condition										
Variance DV L1	0.79***	0.06	0.79***	0.06	0.60***	0.08	0.60***	0.08	0.59***	0.08
Variance DV L2	1.24***	0.30	1.18***	0.29	1.19***	0.32	0.59†	0.30	0.59†	0.31
Variance DV L3	0.05	0.25	0.06	0.25	0.13	0.27	0.05	0.21	0.05	0.20
Variance random L2					0.51	0.38	0.54	0.38	0.11	0.38
Variance random L3					0.07	0.28	0.02	0.27	0.02	0.26
Variance group							1.34*	0.57	1.28*	0.61
AIC	1149.249		1152.211		1152.39		1143.176		1138.696	
BIC	1164.760		1183.233		1198.92		1201.342		1204.617	

Supplementary Table 31. *Continued*.

	Model 6		Model 7		Model 8	
	B	SE	B	SE	B	SE
Intercept	4.07***	0.27	3.90***	0.27	3.88***	0.29
T1 vs T2	-0.04	0.12	-0.04	0.13	-0.04	0.13
T1 vs T3	-0.12	0.15	0.01	0.33	0.06	0.39
Group (0 = R 1 = L)	-0.46	0.26	-0.16	0.41	-0.12	0.45
Condition (0 = C 1 = E)	-0.03	0.27	0.25	0.31	0.29	0.37
Group × Condition			-0.45	0.52	-0.53	0.57
T1 vs T2 × Group						
T1 vs T2 × Condition						
T1 vs T2 × Group × Condition						
T1 vs T3 × Group			-0.12	0.28	-0.23	0.42
T1 vs T3 × Condition			-0.13	0.26	-0.24	0.46
T1 vs T3 × Group × Condition					0.20	0.55
Variance DV L1	0.74***	0.10	0.74***	0.11	0.69***	0.10
Variance DV L2	1.26***	0.31	0.56†	0.30	0.59†	0.32
Variance DV L3	0.07	0.29	0.05	0.20	0.04	0.20
Variance random L2			0.09	0.32	0.03	0.39
Variance random L3			0.07	0.21	0.02	0.27
Variance group			1.34*	0.57	1.33*	0.63
AIC	1158.320		1149.707		1149.748	
BIC	1204.853		1207.873		1215.670	

Note. $ICC_{Level\ 3}$ = 0.027, $ICC_{Level\ 2}$ = 0.591. ****p* <.001; ***p* < .01, *, *p* <.05, †*p* <.10.

Supplementary Table 32. Full results for empathic concern.

	Model 1		Model 2		Model 3		Model 4		Model 5	
	B	SE	B	SE	B	SE	B	SE	B	SE
Intercept	5.59***	0.10	5.25***	0.20	5.23***	0.21	5.27***	0.22	5.26***	0.23
T1 vs T2			0.41***	0.09	0.41***	0.13	0.16	0.22	0.18	0.25
T1 vs T3			0.17	0.11	0.17	0.11	0.17	0.11	0.17	0.12
Group (0 = R 1 = L)			0.05	0.18	0.06	0.19	0.08	0.25	0.10	0.27
Condition (0 = C 1 = E)			0.24	0.21	0.27	0.22	0.27	0.31	0.29	0.32
Group × Condition							-0.22	0.42	-0.26	0.47
T1 vs T2 × Group							0.26	0.23	0.20	0.33
T1 vs T2 × Condition							0.23	0.24	0.18	0.41
T1 vs T2 × Group × Condition									0.10	0.49
T1 vs T3 × Group										
T1 vs T3 × Condition										
T1 vs T3 × Group × Condition										
Variance DV L1	0.62***	0.04	0.58***	0.04	0.56***	0.06	0.56***	0.08	0.56***	0.08
Variance DV L2	0.56***	0.13	0.58***	0.14	0.57**	0.21	0.59**	0.26	0.60*	0.26
Variance DV L3	0.06	0.09	0.05	0.11	0.11	0.12	0.04	0.14	0.04	0.14
Variance random L2					0.02	0.28	0.02	0.33	0.02	0.33
Variance random L3					0.11	0.12	0.01	0.18	0.01	0.23
Variance group							0.08	0.25	0.07	0.29
AIC	1018.141		1007.524		1014.266		1016.649		1020.616	
BIC	1033.652		1038.546		1060.798		1074.815		1086.537	

Supplementary Table 32. ***Continued*****.**

	Model 6		Model 7		Model 8	
	B	SE	B	SE	B	SE
Intercept	5.25***	0.21	5.19***	0.21	5.20***	0.22
T1 vs T2	0.41***	0.10	0.41***	0.10	0.41***	0.11
T1 vs T3	0.17	0.13	0.16	0.19	0.12	0.21
Group (0 = R 1 = L)	0.05	0.19	0.20	0.25	0.17	0.29
Condition (0 = C 1 = E)	0.24	0.23	0.32	0.31	0.29	0.33
Group × Condition			-0.22	0.43	-0.17	0.49
T1 vs T2 × Group						
T1 vs T2 × Condition						
T1 vs T2 × Group × Condition						
T1 vs T3 × Group			-0.10	0.22	-0.02	0.35
T1 vs T3 × Condition			0.11	0.28	0.18	0.36
T1 vs T3 × Group × Condition					-0.15	0.52
Variance DV L1	0.58***	0.05	0.58***	0.05	0.57***	0.05
Variance DV L2	0.59***	0.16	0.55**	0.17	0.55**	0.18
Variance DV L3	0.06	0.17	0.05	0.14	0.05	0.14
Variance random L2	0.02	0.18	0.02	0.23	0.01	0.31
Variance random L3	0.004	0.19	0.003	0.19	0.003	0.20
Variance group			0.07	0.27	0.07	0.28
AIC	1015.844		1020.542		1024.412	
BIC	1062.377		1078.708		1090.402	

Note. DV: Emp $ICC_{Level\ 3}$ = 0.069, $ICC_{Level\ 2}$ = 0.433. ****p* <.001; ***p* < .01, *, *p* <.05, †*p* <.10.

Supplementary Table 33. Full results for perspective taking (2-item scale).

	Model 1		Model 2		Model 3		Model 4		Model 5	
	B	SE	B	SE	B	SE	B	SE	B	SE
Intercept	4.50***	0.12	4.14***	0.22	4.15***	0.23	4.26***	0.26	4.27***	0.26
T1 vs T2			0.28*	0.13	0.28*	0.14	0.17	0.25	0.13	0.33
T1 vs T3			0.12	0.11	0.12	0.12	0.11	0.12	0.11	0.13
Group (0 = R 1 = L)			0.45*	0.22	0.45*	0.22	0.28	0.33	0.26	0.34
Condition (0 = C 1 = E)			0.004	0.25	-0.02	0.27	-0.22	0.35	-0.25	0.35
Group × Condition							0.33	0.44	0.37	0.46
T1 vs T2 × Group							0.01	0.23	0.11	0.38
T1 vs T2 × Condition							0.19	0.28	0.28	0.39
T1 vs T2 × Group × Condition									-0.18	0.48
T1 vs T3 × Group										
T1 vs T3 × Condition										
T1 vs T3 × Group × Condition										
Variance DV L1	0.69***	0.04	0.67***	0.04	0.66***	0.08	0.66***	0.08	0.66***	0.08
Variance DV L2	0.91***	0.22	0.82***	0.20	0.76***	0.28	0.72*	0.32	0.73*	0.33
Variance DV L3	0.07	0.14	0.12	0.15	0.11	0.17	0.12	0.16	0.12	0.16
Variance random L2					0.02	0.28	0.02	0.28	0.02	0.33
Variance random L3					0.01	0.23	0.004	0.23	0.004	0.23
Variance group							0.07	0.44	0.06	0.44
AIC	1087.642		1083.087		1090.285		1094.604		1098.425	
BIC	1103.153		1114.109		1136.818		1152.770		1164.347	

Supplementary Table 33. *Continued*.

	Model 6		Model 7		Model 8	
	B	SE	B	SE	B	SE
Intercept	4.14***	0.24	4.21***	0.27	4.23***	0.28
T1 vs T2	0.28*	0.13	0.28*	0.14	0.28*	0.14
T1 vs T3	0.12	0.12	0.15	0.25	0.10	0.30
Group (0 = R 1 = L)	0.45*	0.23	0.35	0.34	0.32	0.35
Condition (0 = C 1 = E)	-0.002	0.29	-0.19	0.34	-0.22	0.35
Group × Condition			0.32	0.47	0.38	0.49
T1 vs T2 × Group						
T1 vs T2 × Condition						
T1 vs T2 × Group × Condition						
T1 vs T3 × Group			-0.18	0.26	-0.08	0.39
T1 vs T3 × Condition			0.11	0.26	0.20	0.39
T1 vs T3 × Group × Condition					-0.19	0.56
Variance DV L1	0.66***	0.07	0.66***	0.06	0.65***	0.07
Variance DV L2	0.81***	0.25	0.77*	0.31	0.77*	0.31
Variance DV L3	0.12	0.18	0.13	0.16	0.13	0.16
Variance random L2	0.01	0.38	0.01	0.35	0.01	0.37
Variance random L3	0.02	0.28	0.01	0.21	0.01	0.22
Variance group			0.08	0.47	0.08	0.47
AIC	1091.134		1095.432		1099.003	
BIC	1137.667		1153.599		1164.924	

Note. $ICC_{Level\ 3}$ = 0.065, $ICC_{Level\ 2}$ = 0.523. ****p* <.001; ***p* < .01, *, *p* <.05, †*p* <.10.

Supplementary Table 34. Full results for perspective taking (item 1).

	Model 1		Model 2		Model 3		Model 4		Model 5	
	B	SE	B	SE	B	SE	B	SE	B	SE
Intercept	3.80***	0.14	3.37***	0.25	3.38***	0.25	3.43***	0.29	3.49***	0.29
T1 vs T2			0.08	0.16	0.08	0.17	-0.17	0.33	-0.39	0.39
T1 vs T3			0.01	0.16	0.01	0.16	0.01	0.16	0.01	0.16
Group (0 = R 1 = L)			0.76**	0.29	0.74*	0.29	0.72†	0.40	0.59	0.41
Condition (0 = C 1 = E)			0.04	0.28	0.03	0.29	0.00	0.39	-0.12	0.38
Group × Condition							-0.09	0.55	0.16	0.58
T1 vs T2 × Group							0.24	0.31	0.68	0.53
T1 vs T2 × Condition							0.24	0.37	0.66	0.47
T1 vs T2 × Group × Condition									-0.85	0.68
0T1 vs T3 × Group										
T1 vs T3 × Condition										
T1 vs T3 × Group × Condition										
Variance DV L1	1.34***	0.10	1.34***	0.11	1.32***	0.20	1.32***	0.19	1.30***	0.19
Variance DV L2	1.65**	0.49	1.36***	0.38	1.29**	0.42	1.30*	0.63	1.31†	0.68
Variance DV L3	0.01	0.26	0.15	0.23	0.19	0.28	0.16	0.27	0.16	0.29
Variance random T L2							0.04	0.50	0.03	0.61
Variance random T L3							0.01	0.32	0.01	0.37
Variance random Group									0.03	1.00
AIC	1321.165		1320.045		1328.220		1332.630		1333.859	
BIC	1336.709		1351.134		1374.853		1390.922		1399.922	

Supplementary Table 34. ***Continued*.**

	Model 6		Model 7		Model 8	
	B	SE	B	SE	B	SE
Intercept	3.37***	0.25	3.32***	0.29	3.26***	0.31
T1 vs T2	0.08	0.17	0.07	0.17	0.07	0.17
T1 vs T3	0.01	0.17	0.06	0.31	0.25	0.41
Group (0 = R 1 = L)	0.76*	0.30	0.85*	0.43	0.98*	0.46
Condition (0 = C 1 = E)	0.04	0.30	0.10	0.38	0.22	0.40
Group × Condition			-0.12	0.52	-0.36	0.55
T1 vs T2 × Group						
T1 vs T2 × Condition						
T1 vs T2 × Group × Condition						
T1 vs T3 × Group			-0.10	0.30	-0.49	0.46
T1 vs T3 × Condition			-0.01	0.32	-0.38	0.50
T1 vs T3 × Group × Condition					0.75	0.66
Variance DV L1	1.32***	0.14	1.33***	0.14	1.31***	0.15
Variance DV L2	1.36**	0.51	1.35†	0.71	1.35†	0.72
Variance DV L3	0.15	0.31	0.15	0.27	0.15	0.27
Variance random L2	0.04	0.51	0.04	0.51	0.03	0.59
Variance random L3	0.02	0.42	0.01	0.38	0.01	0.38
Variance group					0.03	0.97
AIC	1328.290		1334.333		1336.231	
BIC	1374.923		1392.625		1402.294	

Note. DV: PersT_1 model1 $ICC_{Level\ 3}$ = .003, $ICC_{Level\ 2}$ = .549. ***p <.001; **p < .01, *, p <.05, †p <.10.

Supplementary Table 35. Full results for perspective taking (item 2).

	Model 1		Model 2		Model 3		Model 4		Model 5	
	B	SE	B	SE	B	SE	B	SE	B	SE
Intercept	2.88***	0.15	2.94***	0.24	2.96***	0.25	2.73***	0.29	2.70***	0.31
T1 vs T2			-0.32*	0.15	-0.32*	0.16	-0.27	0.24	-0.22	0.27
T1 vs T3			0.05	0.12	0.05	0.12	0.05	0.13	0.05	0.13
Group (0 = R 1 = L)			0.28	0.19	0.26	0.18	0.70*	0.33	0.75†	0.41
Condition (0 = C 1 = E)			-0.23	0.24	-0.23	0.25	0.17	0.36	0.22	0.39
Group × Condition							-0.77†	0.44	-0.88	0.54
T1 vs T2 × Group							-0.07	0.23	-0.17	0.34
T1 vs T2 × Condition							-0.03	0.25	-0.12	0.37
T1 vs T2 × Group × Condition									0.20	0.54
0T1 vs T3 × Group										
T1 vs T3 × Condition										
T1 vs T3 × Group × Condition										
Variance DV L1	0.82***	0.05	0.78***	0.10	0.72***	0.09	0.72***	0.09	0.72***	0.10
Variance DV L2	0.86***	0.21	0.85***	0.23	1.08***	0.29	0.99**	0.30	0.99**	0.31
Variance DV L3	0.01	0.20	0.01	0.22	0.02	0.27	0.01	0.23	0.01	0.24
Variance random T L2					0.16	0.34	0.16	0.34	0.16	0.40
Variance random T L3					0.03	0.20	0.01	0.18	0.01	0.18
Variance random Group							0.09	0.30	0.08	0.36
AIC	1130.726		1127.735		1122.862		1124.341		1128.135	
BIC	1146.270		1166.596		1169.495		1182.632		1194.199	

Supplementary Table 35. ***Continued*****.**

	Model 6		Model 7		Model 8	
	B	SE	B	SE	B	SE
Intercept	2.97***	0.23	2.80***	0.29	2.81***	0.29
T1 vs T2	-0.32*	0.13	-0.32*	0.13	-0.32*	0.13
T1 vs T3	0.05	0.16	-0.11	0.25	-0.19	0.27
Group (0 = R 1 = L)	0.26	0.20	0.63†	0.33	0.60†	0.34
Condition (0 = C 1 = E)	-0.26	0.25 s	0.10	0.36	0.07	0.38
Group × Condition			-0.78†	0.44	-0.72	0.48
T1 vs T2 × Group						
T1 vs T2 × Condition						
T1 vs T2 × Group × Condition						
T1 vs T3 × Group			0.13	0.24	0.29	0.33
T1 vs T3 × Condition			0.19	0.25	0.34	0.32
T1 vs T3 × Group × Condition					-0.31	0.44
Variance DV L1	0.70***	0.08	0.70****	0.08	0.70***	0.09
Variance DV L2	0.75**	0.25	0.68*	0.31	0.69*	0.33
Variance DV L3	0.01	0.27	0.01	0.21	0.01	0.21
Variance random L2	0.15	0.25	0.15	0.27	0.12	0.31
Variance random L3	0.10	0.19	0.09	0.20	0.09	0.20
Variance group			0.08	0.35	0.08	0.37
AIC	1125.843		1126.515		1129.974	
BIC	1172.476		1184.806		1196.038	

Note. DV: PersT_2R $ICC_{Level\ 3}$ = .007, $ICC_{Level\ 2}$ = .508. ****p* <.001; ***p* < .01, *, *p* <.05, †*p* <.10.

Supplementary Table 36. Full results for perspective taking (item 3).

	Model 1		Model 2		Model 3		Model 4		Model 5	
	B	SE	B	SE	B	SE	B	SE	B	SE
Intercept	5.20***	0.14	4.92***	0.23	4.92***	0.24	5.11***	0.24	5.08***	0.24
T1 vs T2			0.48***	0.14	0.48**	0.18	0.50	0.36	0.61	0.45
T1 vs T3			0.22	0.14	0.22	0.14	0.22	0.15	0.22	0.15
Group (0 = R 1 = L)			0.12	0.25	0.13	0.25	-0.22	0.33	-0.16	0.42
Condition (0 = C 1 = E)			-0.02	0.25	-0.04	0.29	-0.46	0.35	-0.40	0.36
Group × Condition							0.78	0.52	0.66	0.59
T1 vs T2 × Group							-0.18	0.33	-0.39	0.59
T1 vs T2 × Condition							0.14	0.32	-0.06	0.35
T1 vs T2 × Group × Condition									0.41	0.77
0T1 vs T3 × Group										
T1 vs T3 × Condition										
T1 vs T3 × Group × Condition										
Variance DV L1	1.09***	0.08	1.03***	0.07	1.00***	0.11	0.99***	0.11	0.99***	0.12
Variance DV L2	0.93**	0.27	0.94**	0.28	0.89**	0.29	0.68†	0.39	0.68†	0.40
Variance DV L3	0.08	0.15	0.08	0.16	0.08	0.19	0.10	0.16	0.10	0.16
Variance random T L2					0.05	0.39	0.05	0.39	0.04	0.42
Variance random T L3					0.07	0.32	0.08	0.29	0.08	0.31
Variance random Group							0.34	0.58	0.34	0.60
AIC	1219.738		1214.099		1221.003		1221.862		1225.099	
BIC	1235.282		1245.187		1267.636		1280.153		1291.163	

Supplementary Table 36. ***Continued*.**

	Model 6		Model 7		Model 8	
	B	SE	B	SE	B	SE
Intercept	4.92***	0.24	5.11***	0.25	5.19***	0.25
T1 vs T2	0.48***	0.14	0.48**	0.15	0.48**	0.15
T1 vs T3	0.22	0.17	0.27	0.27	0.02	0.31
Group (0 = R 1 = L)	0.13	0.28	-0.19	0.35	-0.35	0.38
Condition (0 = C 1 = E)	-0.03	0.26	-0.51	0.33	-0.66†	0.34
Group × Condition			0.82	0.62	1.12	0.69
T1 vs T2 × Group						
T1 vs T2 × Condition						
T1 vs T2 × Group × Condition						
T1 vs T3 × Group			-0.33	0.28	0.17	0.44
T1 vs T3 × Condition			0.22	0.33	0.71†	0.37
T1 vs T3 × Group × Condition					-0.98	0.63
Variance DV L1	1.01***	0.10	1.00***	0.11	0.98***	0.11
Variance DV L2	0.93**	0.35	0.71†	0.37	0.71†	0.37
Variance DV L3	0.09	0.21	0.12	0.16	0.12	0.15
Variance random L2	0.05	0.57	0.04	0.55	0.03	0.55
Variance random L3	0.04	0.40	0.03	0.32	0.03	0.31
Variance group			0.29	0.63	0.29	0.63
AIC	1222.351		1220.507		1219.800	
BIC	1268.984		1278.799		1285.864	

Supplementary References

British Politics at Queens (2024). One PM too many? Brexit, Party gate, and the fall of the Conservative Government. https://blogs.qub.ac.uk/pb-happ/2024/03/06/one-pm-too-many-brexit-party-gate-and-the-fall-of-the-conservative-government/

Butler, P. (2021). British leavers and remainers as polarised as ever, survey finds. https://www.theguardian.com/politics/2021/oct/21/british-leavers-and-remainers-as-polarised-as-ever-survey-finds

Davis, M. H. Measuring individual differences in empathy: Evidence for a multidimensional approach. *J. Pers. Soc. Psychol.* **44**, 113–126 (1983).

Diener, E., Emmons, R. A., Larsen, R. J., & Griffin, S. The satisfaction with life scale. *J. Pers. Assess.* **49**, 71-75 (1985).

Fischer, A. H. & Roseman, I. J. Beat them or ban them: The characteristics and social functions of anger and contempt. *J. Pers. Soc. Psychol.* **93**, 103–115 (2007).

Hox, J. J., Moerbeek, M., Schoot, R. *Multilevel analysis: Techniques and applications*. (2018).

Muthén, L. K., Muthén, B. O. Mplus user's guide (8th ed.). (1998–2017).

National Centre for Social Research, (2020). The British Social Attitudes survey https://lgiu.org/briefing/natcen-british-social-attitudes-survey-2020/

National Centre for Social Research (2024) Identity issues now a key dividing line in Britain's politics. https://natcen.ac.uk/news/identity-issues-now-key-dividing-line-britains-politics

Pauketat, J. V., Mackie, D. M., & Tausch, N. Group-based meta-emotion and emotion responses to intergroup threat. *Br. J. Soc. Psychol.* **59**(2), 494-521 (2020).

Popan, J. R. et al. Political groups in contact: The role of attributions for outgroup attitudes in reducing antipathy. *Eur. J. Soc. Psychol.* **40**, 86–104 (2010).

Prike, T., Reason, R., Ecker, U. K., Swire-Thompson, B., & Lewandowsky, S. (2023). Would I lie to you? Party affiliation is more important than Brexit in processing political misinformation. Royal Society Open Science, 10(2), 220508. https://doi.org/10.1098/rsos.220508

Pew Research Center (2019). Brexit divides the UK, but partisanship and ideology are still key factors https://www.pewresearch.org/short-reads/2019/10/28/brexit-divides-the-uk-but-partisanship-and-ideology-are-still-key-factors/

Renger, D. & Reese, G. From Equality-Based Respect to Environmental Activism: Antecedents and Consequences of Global Identity. *Polit. Psychol.* **38**, 867-879 (2017).

Schriber, R. A., Chung, J. M., Sorensen, K. S., & Robins, R. W. Dispositional contempt: A first look at the contemptuous person. *J. Pers. Soc. Psychol.* **113**(2), 280-309 (2017).

Stewart-Brown, S., Tennant, A., Tennant, R., Platt, S., Parkinson, J., & Weich, S. Internal construct validity of the Warwick-Edinburgh Mental Well-being Scale (WEMWBS): a Rasch analysis using data from the Scottish Health Education Population Survey. *Health Qual. Life Outcomes* **7**(1), 15 (2009).

The Policy Institute King's College London (2019). How polarised is the 2019 general election? https://www.kcl.ac.uk/policy-institute/assets/how-polarised-is-the-2019-general-election.pdf

The Policy Institute King's College London (2022). Leavers 'more likely to regret Brexit vote' than Remainers, study finds. https://www.kcl.ac.uk/news/leavers-more-likely-to-regret-brexit-vote-than-remainers-study-finds

The Week Staff. (February 16, 2023 Thursday). Brexit: what changed after the UK pulled out of the EU. The Week. https://advance.lexis.com/api/document?collection=news&id=urn:contentItem:67JT-R5X1-JB34-4239-00000-00&context=1519360.

Willis, J., Daniels, M., Disler, G., Khalil, L., & Zhou, A. Reliability and Validity of the Intergroup Compromise Inventory in Two Bipartisan Samples. *SAGE Open* **7**(4), 215824401773933 (2017).

YouGov (2022). One in five who voted for Brexit now think it was the wrong decision. https://yougov.co.uk/politics/articles/44445-one-five-who-voted-brexit-now-think-it-was-wrong-d?redirect_from=%2Ftopics%2Fpolitics%2Farticles-reports%2F2022%2F11%2F17%2Fone-five-who-voted-brexit-now-think-it-was-wrong-d
